# The Impact of Cooperation Under Climate Constraints: An Agent-Based Model for Exploring Paleolithic Behavioral Adaptations in the Inner Asian Mountain Corridor

**DOI:** 10.1007/s10816-025-09739-z

**Published:** 2025-09-20

**Authors:** María Coto-Sarmiento, Abay Namen, Aristeidis Varis, Radu Iovita

**Affiliations:** 1 Social Resilience Lab, Aarhus University, Aarhus, Denmark; 2 Department of Sociology and Anthropology, School of Sciences and Humanities, Nazarbayev University, Astana, Kazakhstan; 3 Department of Geosciences, Early Prehistory and Quaternary Ecology Research Group, University of Tübingen, Tübingen, Germany; 4 Department of Geosciences, Institute of Archaeological Sciences/Paleoanthropology Research Group, University of Tübingen, Tübingen, Germany; 5 Center for the Study of Human Origins, Department of Anthropology, New York University, New York, NY USA; 6 HUMANE, Human Ecology and Archaeology Research Group, Department of Archaeology and Anthropology, Milà i Fontanals Institution for Research and Humanities, IMF-CSIC, Barcelona, Spain; 7 Centre for Urban Network Evolutions, Aarhus University, Aarhus, Denmark; 8 Department of Earth Sciences, University of Oxford, Oxford, UK

**Keywords:** Pleistocene, Central Asia, Behavioral adaptations, Evolutionary model, Human cooperation, Agent-based model

## Abstract

Modern humans dispersed throughout the entire world during the Pleistocene, completing an important part of our evolutionary history. Central Asia, one of the most challenging territories to be colonized, is characterized by continental climate and stark geographic contrasts and therefore offers an ideal context for testing hypotheses about the role of human behavior in dispersals under adverse conditions. Here, we introduce an evolutionary theoretical agent-based model exploring the effects of cooperation on dispersal under different climate constraints in two study sub-regions, the Altai and Tian Shan Mountains. The model uses an evolutionary framework to test cooperation dilemmas in four theoretical climate scenarios based on the average temperature during glacial and interglacial periods. We show that (a) population size can significantly influence the pressure on the group, such that a larger population implies more pressure to cooperate; (b) cooperative behaviors are needed for survival in the harshest conditions; and (c) if the initial probability of non-cooperation is higher, then human groups will tend to be non-cooperative even if a cooperative subgroup pushes them to cooperate. Our results demonstrate that the degree of cooperation significantly impacts survival during periods of extreme climatic deterioration. This work provides valuable insights into the mechanisms influencing the settlement of climatically challenging regions by prehistoric groups.

**Supplementary Information:**

The online version contains supplementary material available at 10.1007/s10816-025-09739-z.

## Introduction

Human dispersal into northern latitudes during the last glacial cycle was an impressive feat of adaptation that remains a subject of discussion. Central Asia, with its cold and arid climates, which were exacerbated by Pleistocene climatic oscillations, presents an ideal laboratory for testing hypotheses about the role of human behavior in dispersal under adverse conditions. Hominins occupied Central Asia continuously since the Lower Paleolithic (Alpysbaev, 1979; Derevianko *et al*., 1998a, 1998b; Finestone *et al*., 2022; Krivoshapkin *et al*., 2020; Ranov, 1995). Archaeological records also demonstrate hominin persistence in Central Asia during the climatic fluctuations of the Late Pleistocene (Derevianko *et al*., 2000; Fitzsimmons *et al*., 2017; Iovita *et al*., 2020; Kolobova *et al*., 2011; Krivoshapkin *et al*., 2007; Ozherelyev *et al*., 2021; Taimagambetov, 1990). The timing and chronology of hominin dispersals across Central Asia were initially built on basic techno-typological traits of lithic assemblages (Ranov & Davis, 1979). The latest models, however, rely on abiotic environmental parameters to predict site distribution during interglacial and glacial periods, as well as the possible dispersal routes (Beeton *et al*., 2014; Glantz *et al*., 2018; Li *et al*., 2019, 2020). Such models proposed that the Central Asian region represented a refugium for hominin populations during the Late Pleistocene glaciations (Beeton *et al*., 2014; Iovita *et al*., 2020). In this context, it is reasonable to assume that survival in a challenging and constantly changing environment required a certain degree of cooperation among the Paleolithic populations of Central Asia. This would be required for hunting large game, gathering food, and building shelters. Such activities would have been facilitated by the development of social and communication skills, as well as by the technological development of tools and weapons.

Another aspect of human behavior during the dynamic events of climate change is seen through the prism of cultural adaptations. Cultural adaptation is a fundamental element contributing to changes in human behavior that facilitate the development of cooperative societies (Boyd & Richerson, 2009). Here, we propose an evolutionary agent-based model to explore the effect of behavioral adaptation and social dynamics in the ever-changing environments of Central Asia. Agent-based models have shown their potential to analyze hypothetical scenarios of behavioral patterns in human dispersal (Axtell *et al*., 2002; Callegari *et al*., 2013; Fajardo *et al*., 2020; Hölzchen *et al*., 2016; Hughes *et al*., 2007; Nikitas & Nikita, 2005; Romanowska *et al*., 2017; Steele, 2009; Wren *et al*., 2014). However, numerous unanswered questions persist regarding human adaptation, including the following: How do humans endure the harshest conditions? What role did social strategies play under varying climatic conditions (*e.g.*, fluctuating temperatures)? Did these strategies enhance human adaptability to extreme conditions? Some research in psychology and animal behavior suggests that ecological stress favors tolerance of others in groups and that temperature fluctuations can result in populations adopting a longer-term strategy for survival, lowering the risk of aggression (Lange *et al*., 2017; Testard *et al*., 2024).

This paper proposes an evolutionary model that assesses the possible effect of cooperation as a social strategy for survival in extreme conditions. The model is exposed to different climate scenarios to understand how extreme conditions can affect the cost of maintaining cooperation and the selection of cooperative behavior in groups. We also explore different climate scenarios with the aim of understanding the importance of selecting a social strategy for human dispersal. Four theoretical climate conditions, from the warmest to the coldest conditions, are applied to a hypothetical landscape consisting of two regions (here, the Altai and Tian Shan Mountains) connected by human dispersal.

## Material and Methods

### Regional Setting

Central Asia plays an important role in understanding early human migrations and the settlement of the inner parts of the Asian continent. Existing paleoanthropological and paleogenetic data suggest the synchronous presence and genetic interaction of three hominin metapopulations, the Neanderthals, Denisovans, and modern humans (Gokcumen, 2019). These findings make this region particularly interesting for studying human evolution and cultural development. Although human skeletal remains have not yet been discovered from Paleolithic sites in Kazakhstan, the possibility that these metapopulations co-inhabited the territory corresponding to modern-day Kazakhstan is high (Boivin *et al*., 2013).

**Fig. 1:**
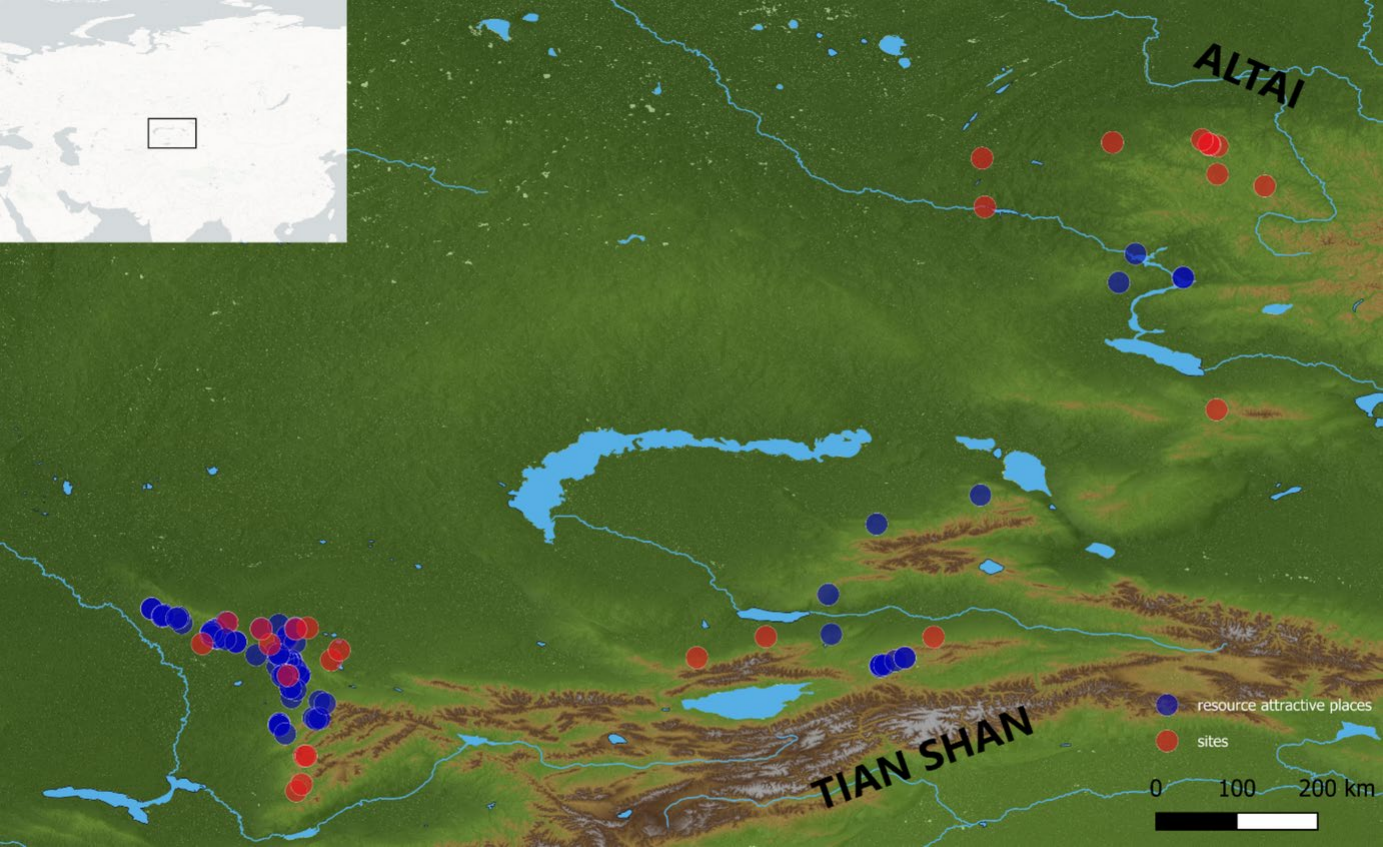
Map of the selected localities within the Altai and Tian Shan regions included in the model. The red dots correspond to the archaeological sites where human presence was detected, while the blue dots correspond to resource-attractive locations

Our study area includes much of the Inner Asian Mountain Corridor (IAMC), a continuous chain of mountain ranges that takes up most of the Central Asian states, Western China, Mongolia, and the Russian Altai (Frachetti, 2012; Frachetti *et al*., 2017). Our fieldwork has concentrated on two key regions of the IAMC, the Tian Shan (southern Central Asia) and the Altai Mountains (northern Central Asia) (see Fig. 1).

The Tian Shan Mountain range is located in the border region between Kazakhstan, Kyrgyzstan, Uzbekistan, and China in Central Asia. The Altai Mountain range, located in northern Central Asia, covers a vast region that is shared by Russia, China, Mongolia, and Kazakhstan.

Within the Tian Shan Mountains, our data derives from several ranges including the Ili Alatau and Qaratau mountains located in Kazakhstan, as well as the Western Tian Shan in Uzbekistan. These regions revealed fossil and archaeological materials dated to the Middle and Upper Paleolithic (Fitzsimmons *et al*., 2017; Glantz *et al*., 2003, 2008; Krivoshapkin *et al*., 2007, 2010; Ozherelyev *et al*., 2019; Taimagambetov, 1990) and therefore of utmost importance to investigate the impact of cooperation under climatic constraints of the Late Pleistocene. The recent intensification of Paleolithic research in the northern foothills of the Tian Shan, particularly in the Ili Alatau range, demonstrates the significance of the region to the study of Late Pleistocene human dispersals and cultural adaptations (Dzhasybaev *et al*., 2018; Fitzsimmons *et al*., 2017, 2018; Namen *et al*., 2024; Ozherelyev *et al*., 2019, 2021, 2023a, 2023b; Taimagambetov & Ozherelyev, 2009).

The Altai Mountains are located in northern Central Asia. The foothills of the Altai have yielded important Pleistocene archaeological sites (Belousova *et al*., 2018; Derevianko *et al*., 1998a, 1998b, 2013), including the Denisova cave that yielded fossil remains of Neanderthals and Denisovans (Brown *et al*., 2022; Douka *et al*., 2019; Jacobs *et al*., 2019; Slon *et al*., 2017, 2018). Even within the last glacial period, climate change affected landscapes differently in different parts of the IAMC (Machalett *et al*., 2008). In particular, the coldest stadial periods were also dry while interstadials were comparatively wetter; mountain glaciers grew in the Tian Shan during periods when it was wet enough, whereas in the Altai, they grew when the temperature was reduced (Koppes *et al*., 2008; Owen & Dortch, 2014).

We seeded our model with the geographical locations drawn from previously published sites and located in the Qaratau, Tian Shan, Dzhungarian Alatau, Tarbagatai Ranges of Tian Shan, and the Altai Mountains. The new survey data were gathered during fieldwork from 2017 to 2022 (Cuthbertson *et al*., 2021; Iovita *et al*., 2020). Our database includes 130 places; 29 correspond to archaeological sites with evidence of Pleistocene and later periods of human occupations in the cave, Rockshelter, spring and open-air geomorphological contexts, and 101 are resource-attractive locations. We define resource-attractive locations as places where Paleolithic humans could have sought shelter. Our database of resource-attractive places includes caves and rockshelters identified during the PALAEOSILKROAD project’s reconnaissance surveys in the Inner Asian Mountain Corridor (Cuthbertson *et al*., 2021).

### Model Design

PaleoCOOP is an agent-based model that simulates human interactions within an evolutionary framework. This model primarily focuses on exploring the impact of behavioral adaptation on the survival of groups of individuals under changing climate and its effect on local environments.

A complete, detailed model description, following the ODD protocol (overview, design concepts, details) (Grimm *et al*., 2010, 2020; Müller *et al*., 2013; Polhill *et al*., 2008) is provided in the “Model Design” section and on the Supplementary Material (ODD protocol). More technical details are available in the “Open Data” section and the Supplementary Materials.

### Purpose

PaleoCOOP provides insights into cooperative behavior, resource competition, and the ability to thrive in challenging climatic conditions by analyzing the interplay between human decision-making and behavioral adaptation. Specifically, we test the influence of significant climatic changes on human cooperation by looking at the relationship between resource availability, cooperation, and group competitiveness.

PaleoCOOP explores four different theoretical climate scenarios, each representing a climate condition glacial and interglacial, with emphasis on either average temperature or seasonal extremes. These scenarios serve as theoretical settings to analyze and compare the adaptive strategies for human cooperation. By examining the effects of climate variation on cooperative behavior, the model enables a comprehensive exploration of the factors that contribute to individuals adopting cooperation or defection in different climatic contexts.

### Environmental Dynamics of the Region

**Fig. 2:**
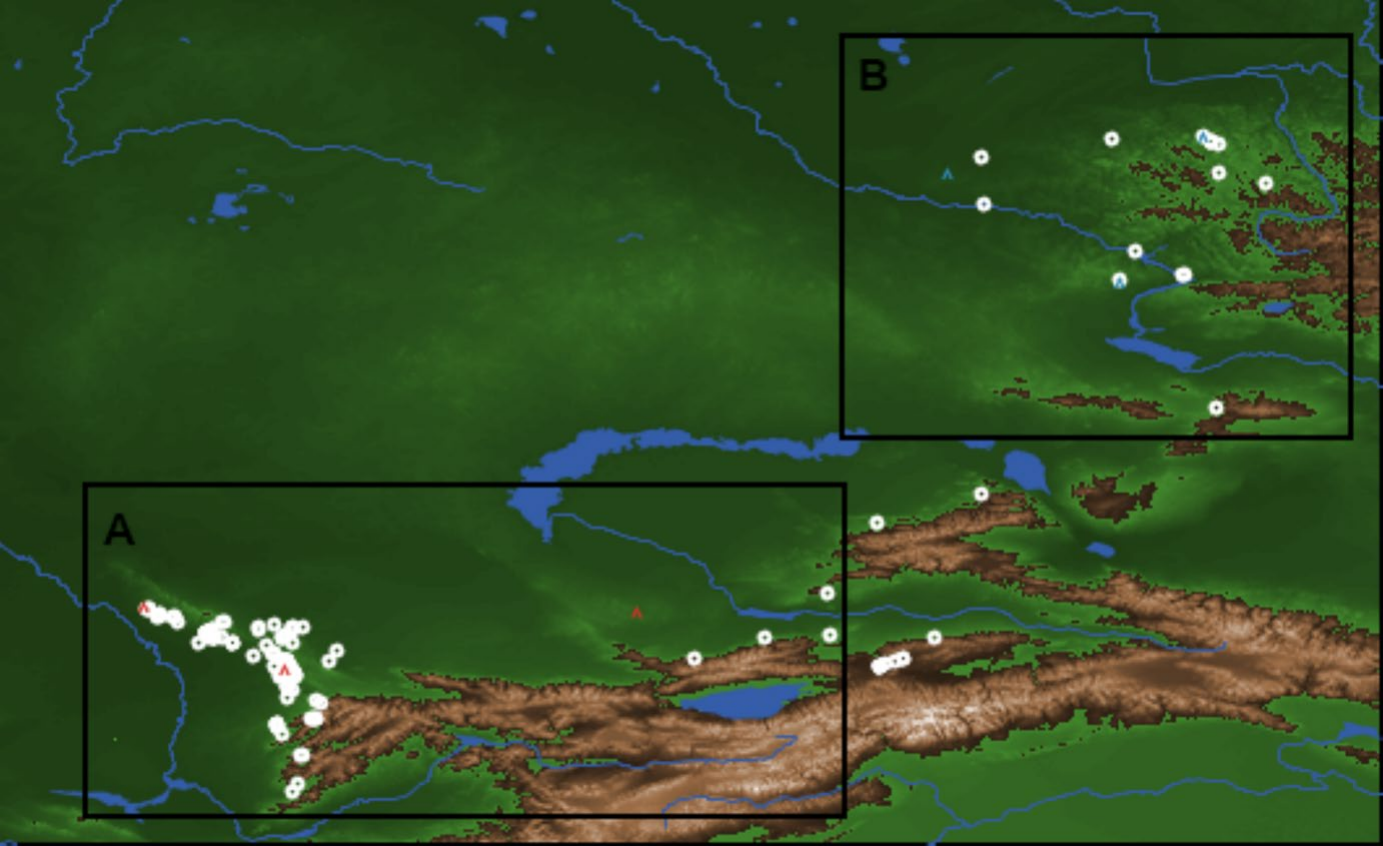
Map of the environment for the model of the Altai and Tian Shan regions (534 × 328). We created two environments for the model: A is located in Tian Shan and B in Altai. White dots represent the archaeological data. Blue and red “houses” are attractor places

The model uses the GIS extension in NetLogo and requires an ASCII elevation raster map. Additionally, the maps use shapefiles with original sites, lakes, and rivers. The map of the environment is represented by a grid of patches (Fig. 2). Water is performed with a shapefile, while an ASCII elevation raster is used to represent mountains.

Patches were divided into two geographical areas—Altai and Tian Shan—within the territory of modern-day Kazakhstan. Each patch on the map Represents 0.5 km^2^ of the geographical area (1 patch size in pixels). Patches are characterized by three key elements: water, mountains, and unspecified resources (*e.g.*, food). Water bodies, including lakes and rivers, represent the existence of water in the environment. Despite the inclusion of water as a component within the model, its substantive role or impact has not been explicitly incorporated or accounted for in fulfilling the objectives of the model. Mountains are defined by their elevation as indicated on the map.

Resources play an important role in the model. The aim here is to establish a theoretical scenario where certain patches have more abundant resources compared to others. This definition of attractiveness is given by the patches with higher resource availability, strategically located in random areas where archaeological sites were found. This approach creates a geographical distribution environment similar to a potential real environment.

Most patches contain Resources that are randomly distributed with a range of 0 to 50, with 50 representing the maximum quantity of resources in these areas. On the other hand, we select attractor patches with a potentially high concentration of resources in specific areas according to the archaeological data provided. However, agents are not restricted to these locations and can also explore intermediate patches, but with lower resource densities. Here, we interpret that the concentration of archaeological sites located in both Altai and Tian Shan could be explained by strategic position and resource availability (Derevianko, 2017; Iovita *et al*., 2020; Varis *et al*., 2022). The patches selected in specific areas start with a Range of 100 as the maximum quantity of resources.

In this context, the model does not account for any discernible correlation between the density of archaeological sites and the abundance of available resources. Archaeological data is only used as a theoretical example to visualize the different availability of resources in patches and the attractiveness of the place, *i.e.*, the attractiveness of resources in certain patches is determined by archaeological data in the model. We therefore prefer to designate the presence of multi-occupation sites as “resource-attractive,” even without being able to specify the exact resources.

Humans consume resources at every step of the simulation, while resources can also be decreased naturally due to climate change. Once resources are non-optimal, every resource patch can gradually regenerate. Only resources in attractor patches can grow, reduce, or regenerate over time, while the rest of the patches do not use this function. This design choice helps to minimize the time cost of the model, assuming that individuals will move toward patches with higher resource availability. Therefore, within this model, patches that are not attractors can only experience modifications in their resources while remaining unchanged in their environment.

A known limitation of our model is that site locations were used to define resource-rich areas, which could introduce a degree of circularity in our approach. However, our goal here is not to predict new site locations but to explore whether cooperation strategies can sustain populations at these known locations under varying climate conditions. Future work could address this limitation by incorporating independent paleoenvironmental reconstructions of resource availability.

We are also aware that research and preservation biases may affect the model, potentially skewing the spatial distribution of the placement of attractor patches. While we recognize that this introduces a degree of uncertainty, we selected this approach rather than attempting to reconstruct resource distributions based on highly incomplete paleoenvironmental data, which would also involve significant assumptions.

It is important to emphasize that we do not interpret the clustering of sites as evidence of actual high resource density. Our goal is not to predict the location of new sites, but rather to test the feasibility of different cooperation strategies in sustaining populations at locations where human presence is attested. In addition, we recognize that the relative magnitude of resource abundance likely influences model outcomes. In future work, alternative scenarios with varying levels of resource differentiation could be explored to assess the robustness of our findings.

### Process Overview and Scheduling

We conduct simulations for four theoretical scenarios, with each time step representing one month (one tick = one month). The simulation spans 1200-time steps (months), the equivalent of 100 years. The initialization and the end of the simulation may differ based on the specific experiment being conducted. The aim is to analyze the effect of climate change on behavioral patterns in both areas of the IAMC, examining changes at both small and large scales within a short period of time frame.

The model incorporates three different entities (agents) from the Tian Shan and Altai, as well as from the different attractor sites. The two groups of humans are initially located in different random locations within the Tian Shan and Altai areas. The specific characteristics of humans from each area are outlined in Table 1. To ensure a more homogeneous model design, both groups of humans possess identical physical and behavioral features to maintain consistency and standardization at the beginning of the simulation.

**Table 1:** Factors of human agents in the model

Factors	Brief description	Default level
nHominins	Defines the number of individuals in the simulation. In the model, hominins are the human agents from Tian Shan and hominins (heminins in the model) from Altai	10
hominin-speed	Defines how individuals move in the environment	0.75 km
learning-rate	Defines the probability of learning the path to arrive someplace	1
human-risk	The risk that human agents perceive. Risk is related to difficult situations to survive	5
max-energy	The energy of the human agents	100
age	Age of human agents. The mortality Rate of individuals is around 50 years	1–50
traits	Define how human agents transmit and spread traits to others. Hominins get one trait when they are born. 1 is to tend to cooperate and 0 is to tend to defect	0 or 1
body-temperature	The average body temperature of human agents is 37 °C	37 °C
scenarios	Theoretical scenarios	Scenario 1 Scenario 2 Scenario 3 Scenario 4

The population size for each group is determined by the experiment, which can be adjusted using the slider. Both Altai and Tian Shan have an equal number of agents at the beginning of the simulation. 

We incorporate “attractors,” which represent places of interest with greater resource availability. Attractors possess their own set of resources that agents close to the attractors can use to shelter and gain energy. In this model, we use the term “attractor” to refer to resource-rich locations that may attract hominin populations due to their ecological advantages, as previously described. This usage differs from the concept of “attractor” in complexity science, where it refers to emergent stable states in dynamic systems (see *e.g.*, Ullah *et al*., 2015). The attractors are randomly placed within the geographical environment, where high resource availability of patches is located although it is independent of these patches. For the model, three static attractors are initially created for each area of Altai and Tian Shan. The number of attractor places can change over time in the simulation, either increasing when new places are founded or decreasing when all the resources in the attractors are spent.

**Fig. 3:**
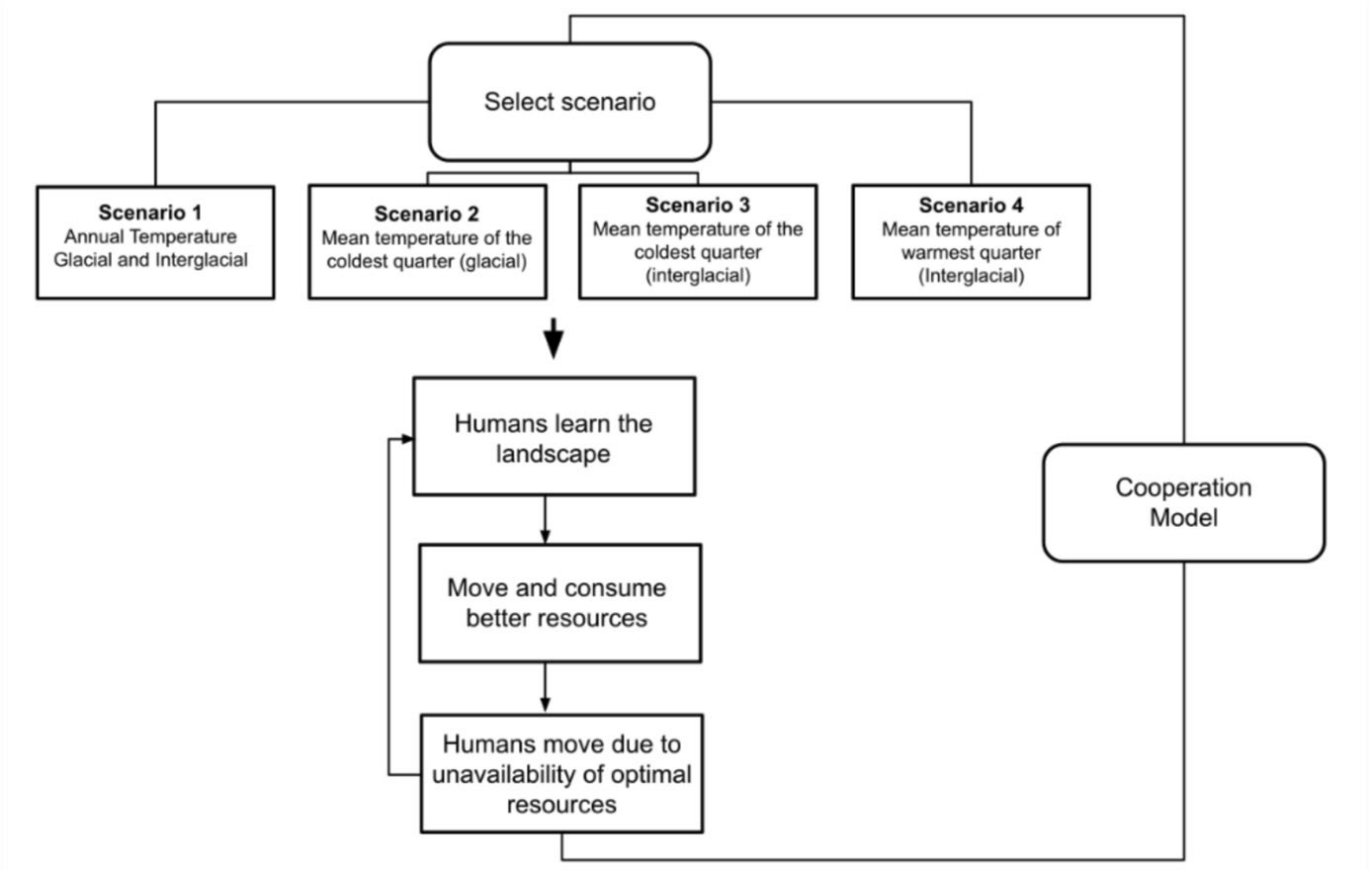
A diagram of the stages for the model

The model develops distinct stages throughout the simulation, which can be observed in Fig. 3. These stages include learning, interaction (cooperation model), resource consumption, and ultimately, moving to another location.

To increase the plausibility of the model, individuals are Randomly assigned varying ages, with an average mortality rate set at approximately 50 years (Trinkaus, 2011). The Reproductive capability is enabled for individuals over the age of 12, with a 10% chance of reproduction rate (Dennell *et al*., 2011; Weiss, 1973). Across the different scenarios, the reproduction rate and average age remain constant. By incorporating these age-related factors and reproductive considerations, the model captures essential aspects of human life cycles and population dynamics, contributing to a more accurate representation of ancient societies.

At the beginning of the simulation, humans can move through the landscape while simultaneously acquiring the knowledge required to identify areas with better resources. We use the human-learning factor to incorporate complexity into how people can learn the proper path to arrive at the attractor locations. Once individuals learn the optimal path to reach a place, they move toward areas with better resources. We assume that better resources are concentrated in specific areas although they may still consume resources all over the land. 

The consumption of resources varies in individuals depending on three factors: (a) the social strategy that individuals choose, (b) the specific scenarios, and (c) the region. In the model, the selection of a cooperation strategy may always imply that individuals consume fewer resources than defectors, as well as in more extreme scenarios where resources are limited. In addition, resource consumption can be affected by the regions where individuals are located, whereas in Altai, the resources can be more limited. While cooperative behavior prioritizes group welfare and resource sharing, defective behavior prioritizes self-interest without considering the well-being of others (Carballo, 2012). The probability of cooperation of the population can be previously selected in the model: a percentage of 0 means no probability of cooperation, while 100% is a full probability of cooperators within the population. The role of cooperation for each individual can change based on the cooperation model during the simulation (see cooperation model subsection).

All individuals begin with a maximum energy level of 100%. The energy level can be adjusted using the max-energy slider, which describes the percentage of the energy of humans. The energy is spent differently, and it will vary depending on the scenarios and the place where humans are (safe vs. non-safe). For example, we assume that individuals are constantly spending energy at each timestep, although the amount of energy spent varies given the perception of the risk and the climate conditions. While energy consumption can be slowed down, it cannot be restored unless they are near attractor places where they can recover energy.

In addition, individuals may also consume extra energy obtained from attractor places. These places not only provide energy but also serve as shelter from extreme cold conditions for the population. In other words, the attractor places contribute to increasing the body temperature and the energy of humans.

The cooperators have the initiative to create new attractors but need to have nearby individuals, both cooperators and non-cooperators, to establish these new attractors. Both cooperators and non-cooperators can benefit from the resources of the attractors equally.

It is assumed here that individual non-cooperators are unable to cooperate to help other individuals fund new sites unless a limited number of cooperators are present. In addition, attractors can be founded under two conditions: (a) attractors must be spaced at least 5 km apart and (b) the patch, where they are located, must have at least 50% of its available resources left. Patches need to be in optimal conditions for the foundation. If neither condition is met, then attractors cannot be founded.

Non-cooperators and defectors can still consume energy from existing attractor locations. However, excessive resource consumption can cause an attractor to disappear. Attractors can be founded again when both conditions occur again.

Energy in individuals is linked to climate and the perceived risk by individuals in their environment. In extreme climate scenarios, individuals need to spend more energy compared to scenarios with less extreme climates.

The rate of energy gain/loss is also influenced by the level of perceived Risk and the distance from attractors. In the model, agents assume a lower risk in areas with more resources or attractor places. When individuals do not perceive a significant risk, their energy decreases more slowly. In locations with Limited resources, higher perceived risk accelerates energy depletion, especially when the risk perception is over a threshold of 80%. It is significant to notice that individuals can perish if they deplete all their energy and the energy level drops to 0. Thus, individuals located in attractor places are expected to expend less energy compared to those in non-attractor places, as we assume that attractor locations provide a sense of safety and security. This difference in energy expenditure between the attractor and non-attractor places is a crucial aspect of the model, as it reflects the adaptive behaviors of individuals seeking out favorable environments for resource acquisition and protection.

The model includes the analysis of mobility strategy patterns and resource availability (Santos *et al*., 2015; Wren and Costopoulos, 2015). We previously defined a place of origin where hominins initially arrive and a second place where humans migrate. Agents can migrate from their place of origin to other locations over long distances if resources are scarce, to find new available resources. Migration occurs when individuals in patches with no resources fail to find available resources within a period of time, forcing them to seek new resources by moving to other areas with more available resources. Moreover, agents leave their place of origin and move to other areas when the resources are not optimal (Belovsky, 1988; Bettinger *et al*., 2015). We assumed here that Resources from patches below 20% are the optimal limit for the full use of resources without the need to exhaust resources before changing location. This threshold indicates that resources are not optimal, prompting individuals to conserve their remaining resources. Thus, some individuals are compelled to migrate to alternative areas with better resource availability.

Upon migrating to a new location, the first stage of the model starts again, continuing until resources again become limited within that area. At such a point, individuals are once again compelled to move, either back to where they came from or to other locations nearby.

Here, the dynamics of the model are designed to explore the effect of dispersal dynamics on the population, specifically examining the role of (a) resource scarcity, (b) cooperation strategy, and (c) climate scenarios. This dynamic illustrates a significant mobility pattern observed in humans when searching and competing for resources distributed in specific locations.

### Submodels

#### Climate Scenarios

The model explores theoretical climate scenarios for each region, based on the average temperature observed during glacial and interglacial periods. The objective is to compare different bioclimatic variables and observe how the mechanisms of human behavior react differently. The selection of these climate temperatures aims to test and compare representative examples of temperatures, both extreme and non-extreme, during the glacial and interglacial periods.

We use the climate estimation proposed by Glantz *et al*. (2018) for the glacial and interglacial periods in the Tian Shan and Altai areas (refer to Table 2 from Glantz *et al*., 2018). Data and temperatures were collected by selecting the Community Climate System Model (CCSM4) for the Last Glacial Maximum (26.5–19 ka) and the last interglacial (MIS 5e ca. 125,000 years ago) (Clark *et al.*, 2009; Otto-Bliesner *et al*., 2006).

In our study, we incorporate seasonality into the model by updating the climate data every three months. We first adopt the climate estimation method proposed by Glantz *et al*. (2018) for the model setup. Then, we select the variables minimum temperature (°C), maximum temperature (°C), and average temperature for each month for both the Altai and Tian Shan regions. To integrate seasonality into the model, we calculate the total mean for each three months to represent the results for every season. Data variables were collected and downloaded from the WordClim dataset (www.worldclim.org) (Fick & Hijmans, 2017; Hijmans *et al*., 2005).

The model performs four distinct theoretical climate scenarios, which can be selected using the chooser in the model interface, as can be seen in Table 2.

**Table 2:** Setup and go of the scenarios with the mean temperatures selected for the model based on Table 2 from Glantz *et al*. (2018) with modification in Scenario 1 by calculating the mean of both glacial and interglacial

**Setup**			
**Scenarios**	**BioClim variables**	**Tian Shan**	**Altai**
1	Annual Mean Temperature (glacial and interglacial)	6 °C	− 4 °C
2	Mean temperature of the coldest quarter (glacial)	− 7 °C	− 21.5 °C
3	Mean temperature of the coldest quarter (interglacial)	− 9.6 °C	− 20 °C
4	Mean temperature of warmest quarter (interglacial)	25 °C	19.4 °C
**Go**			
**Scenarios**	**Seasonality**	Tian Shan	Altai
1	Winter	− 0.98	− 16.4
	Spring	11.26	2.60
	Summer	24.42	19.37
	Autumn	12.35	3.97
2/3	Winter	− 7	− 20
	Spring	7.29	− 4.42
	Summer	19.20	11.54
	Autumn	4.95	− 2.92
4	Winter	3.67	− 10.05
	Spring	16.64	9.83
	Summer	31.59	27.18
	Autumn	18.76	10.88

In addition to the climate temperature, the scenarios differ in the behavioral patterns of humans, specifically in terms of energy expenditure, body temperature, and resource consumption. However, the cooperation model remains without any changes in all the scenarios.

The scenarios have been specifically designed to explore the potential behavioral response of humans in an environment with variable climate temperatures. Due to the divergence in the climate temperature in the two areas, Altai and Tian Shan are represented differently, with the Altai area having more extreme climate conditions than the Tian Shan area.

- *Scenario 1* uses the total of the means of annual temperature in °C for glacial and interglacial scenarios. Since there is not a large temperature difference between glacial and interglacial temperatures, we combine both periods within the model and compute the mean for each scenario. In this scenario, the environment is milder compared to the extreme scenarios, resulting in reduced mental (risk perceived) and physical costs (energy) associated with human learning to find resources. Here, climate and environment are less hostile to human mobility. The difference in consumption will be given by the area and the social strategy taken. Additionally, humans also expend the least amount of energy during movement compared to other scenarios.
- *Scenario 2* uses the mean temperature of the coldest quarter in the Glacial Scenario. Unlike the previous scenario, this scenario presents a higher survival cost due to the extremely cold temperature. The increased costs for humans in this scenario can be attributed to two variables: (a) higher energy expenditure: the environment is more costly for human dispersal, leading to increased energy expenditure as individuals strive to survive and compete for limited resources and (b) extended exposure to extreme temperatures: humans spend more time in locations where the temperature is more extreme, adding to their survival challenges.
- *Scenario 3* is represented by an interglacial scenario, characterized by the mean temperature of the coldest quarter. This scenario shares similar features with the previous scenario and only differs in the temperature.
- *Scenario 4* is Represented by an interglacial scenario characterized by the mean temperature of the warmest quarter. We select to focus only on the interglacial temperature period since the difference between glacial and interglacial temperatures, particularly for the warmest periods, is not substantial. According to the temperature, the cost of survival in this scenario is comparable to Scenario 1. However, we introduce limited Resources, simulating an extremely arid environment with scarce available resources to test differences between Scenario 1 and Scenario 2.

#### Body Temperature

Climate temperature is defined as distinct temperature environments for each area and scenario. While the patches in both Altai and Tian Shan share similarities in terms of resources and geographical backgrounds in the model, the temperature component is defined according to the climate temperature variability specific to each region. To do so, the grid of patches is divided into two parts to create two approximate theoretical environments with different climate temperatures for each scenario.

Temperature significantly affects individuals in the model. Humans are assigned a body temperature of 37 degrees, according to the average normal body temperature (Petrone *et al*., 2014). In extreme scenarios, humans who remain in unsafe locations for prolonged periods are at risk of hypothermia and eventual death. Safe places with suitable climate conditions are considered attractors to mitigate the effects of cold temperatures. These serve as a refuge, allowing individuals to maintain a stable body temperature and energy. Each time that individuals occupy such an attractor, their body temperature increases by one degree per step, indicating the warming effect of the safe environment.

We also calculate the loss of body temperature:

1 $$\Delta {T}_{b} =({T}_{b }-{T}_{c} ) \cdot CR$$

where $\Delta {T}_{b}$ is the result of the difference between the previous and the present body temperature for each time-step, ${T}_{b}$ is the body temperature at a given time-step, ${T}_{c}$ is the climate temperature for each scenario in Altai or Tian Shan. For the climate temperature, we use the climate estimations suggested by Glantz *et al*. (2018) for the Tian Shan and Altai regions (see “scenarios” subsection). The $CR$ or *cooling-rate* defines the body temperature loss rate, and in that sense is defined differently in the more extreme scenarios. It is important to highlight that when an individual’s body temperature drops below 30 degrees, they are at critical risk of hypothermia and death.

#### Cooperation

Cooperation represents one of the most common phenomena adopted by different species. We follow the definition of cooperation as cultural behavior that provides a positive outcome to an individual or group or that is beneficial for both (Dale *et al*., 2020; Sachs *et al*., 2004).

Cooperative behaviors, such as helping others, the act of assistance, or several people working together to achieve a common goal, play a crucial role in human society (Axelrod, 1984; Axelrod & Hamilton, 1981; Boyd & Richerson, 2009; Melis & Semmann, 2010). While some strategies require a significant cost in terms of individual fitness, cooperation is widely regarded as an adaptive and beneficial behavior that promotes collective success and enhances the overall well-being of society (Carballo, 2012).

In our model, cooperation refers to the behavior of individuals that contributes to the well-being and survival of the group, even when it comes at a potential personal cost. It could be in the form of resource sharing, collective movement, and/or group hunting. This means that a cooperating individual engages in actions that might temporarily reduce their own resources, energy, or well-being, but which ultimately benefit the collective survival of the group.

Cooperation can be maintained and controlled using many different social mechanisms, including punishment, solidarity, reciprocity, and reputation, among others (Axelrod, 1984; Bowles & Gintis, 2004; Boyd *et al*., 2010; Carballo, 2012; Henrich & Henrich, 2007; Rand & Nowak, 2013). They are acquired and transmitted through social learning and payoff-based adaptations. In this case, cooperation is advantageous under low-resource environments or even during high predation risks. This definition would ensure that cooperation is not assumed but rather arises as a testable hypothesis because the implementation of a framework where agents tend to cooperate or defect with payoffs determined by resource availability. This allows us to understand the role of cooperation in dispersal under climatic oscillations of the Late Pleistocene.

We hypothesize that, in challenging circumstances, such as during periods of extreme climate seasonality caused by climate change, cooperation becomes crucial for survival (Aktipis *et al*., 2016; Cornwallis *et al*., 2017; De Jaegher, 2017; Liu & Chen, 2018; Martin *et al*., 2020). This presents an opportunity to examine the dynamics of cooperative behavior in the face of uncertainty. For instance, societies may overcome challenges, such as requiring individuals to balance individual gains with the preservation of collective resources or devise and enforce punishment mechanisms to discourage defectors who undermine collaborative efforts necessary for the group’s survival.

This evolutionary model is inspired by the work of Henrich and Boyd (Henrich, 2001; Henrich & Boyd, 2001) who explored cooperation dilemmas. For these authors, the mechanism of conformist transmission allows cooperation to be maintained without the need for extensive punishment. In other words, the cost of cooperation does not require a high punishment rate to stabilize the population when the population adopts a common behavior (Henrich & Boyd, 2001: 81).

The equation of the cooperation model can be described as follows (Henrich & Boyd, 2001):

2 $$\Delta{p_o=p_o(1-p_o)\lbrack(1-\alpha)\beta(b_c-b_d)+\alpha(2p_o-1)\rbrack}$$

where the variable $\Delta p_o$ represents the change of frequency of cooperators in the population; $p_o$ is the frequency of cooperators, $\beta$ is a normalization parameter, the parameter alpha (*α*) is the strength of the conformist transmission; (${b}_{c } - {b}_{d}$) are the payoff of cooperators and defectors.

${b}_{c } - {b}_{d}$ is given by the following equation:

$$b_c=(1-e)(p_oB(1-e)-C+e(p_oB-Np_1\rho)),$$

$$b_d=(1-e)(p_oB-Np_1\rho),$$

3 $$\Delta b_o=b_c-b_d=(1-e)(Np_1(1-e)\rho-C).$$

where $N$ is the number of individuals; ${b}_{c}$ is the payoff of cooperation while ${b}_{d}$ is the payoff of defectors; *e* is the probability of failing in cooperation; *C* is the cost that cooperators assume to contribute; $B$ is the benefit divided for all groups, and $\rho$ is the punishment.

Henrich and Boyd’s model was not used to define cooperation in the model *per se* on a population scale level, but rather to explore how cooperation might spread within a population through conformist transmission. In our model, cooperation is an agent-level decision based on individual payoffs and local interactions, rather than being directly dictated by population-level dynamics.

Our model incorporates the cooperation strategy based on three key premises: (a) the evolutionary framework proposed by the evolutionary model, (b) the probability of individuals cooperating, not cooperating or defecting, and (c) the distribution and consumption of resources depending on the strategy chosen.

A detailed list of factors for our cooperation model can be listed in Table 3.

**Table 3:** Factors for the cooperation model. Some factors are included in the equation proposed by Henrich and Boyd (2001)

Factors	Overview description
cooperate?	Defines if individuals are cooperators or non-cooperators/defectors. True if agents cooperate. False if humans do not cooperate Cooperation is distinguished by colors: agents with green color are cooperators and red color are defectors. It is defined as a boolean (true or false)
cooperative?	Count agents with cooperation true.
no cooperative?	Count agents with cooperation false.
prob-cooperation	Defines the probability of cooperation for agents at the beginning of the simulation. Default level: 0.20
cost-cooperation	Defines the cost of each cooperator that Reverberates similarly among the group. No cooperators do not pay the cost but they benefit equally. Default level: 10
punishment-cooperation	Defines punishment of cooperation in defectors. Default level: 7
prob-nocoop	Defines the probability of no cooperation for humans. It should be a small number. Default level: 0.001
alpha	Strength of conformist transmission. Range from 0 to 1. Default level: 0.20
pay-off tienshan	The pay-off of the population in Tian Shan
pay-off altai	The pay-off of population in Altai
delta-p	Rate of change of cooperative population
radius-for-strategy	The scope of the cooperation strategy will depend on the number of cooperators surrounding the agent. Default level: 3

To enhance visibility and distinguish the roles of each individual in the model, cooperators are represented by the color green, while non-cooperators and defectors are listed by the color red. Despite the differences between non-cooperators and defectors, their impact on resource allocation and group dynamics can overlap in practice, making it difficult to clearly distinguish their roles within the model’s framework. New individuals below the age of 12 are visually Represented in black, and once they reach 12, the color changes to indicate whether they become cooperators or defectors. This age-dependent color transition reflects the idea that the possibility of choosing a different strategy role is also influenced by age. Although several studies have detected cooperation strategies in children from a very early age (Slocombe & Seed, 2019), the selection of a higher age corresponds to the possible absence of full development of cognitive and strategic abilities in selecting cooperative or non-cooperative decisions (Gutiérrez-Roig *et al*., 2014). In our model, the selection of one strategy is primarily focused on the pressure of the group, instead of being conditioned by their own experience, since they are able to make decisions. Social pressure works similarly to real-life scenarios: if there is a high number of individuals around who are inclined to cooperate, a neutral individual is more likely to choose cooperation as well.

At the beginning of the simulation, humans may assume either role randomly depending on the probability of cooperation that can be set up previously. Within the model, humans interact with others to adopt one of the two social strategies: cooperation and non-cooperation. When humans choose to cooperate, they assume trait 1, whereas non-cooperators assume trait 0.

The assumption of one strategy is linked to the way of consuming resources in the model. By choosing to cooperate, they may adopt strategies such as resource conservation, equitable distribution, or sustainable resource management. These actions lead to more efficient utilization of resources, reducing wastage or overconsumption. In contrast, defectors prioritize their self-interest and may engage in competitive behaviors, seeking to maximize their personal gains without considering the well-being of others. Consequently, defectors may exhibit higher rates of resource consumption as they prioritize their immediate needs or personal accumulation without regard for long-term sustainability or equitable distribution. Therefore, in the model, the selection of a cooperative strategy leads to individuals consuming fewer resources as they prioritize sustainable resource use and equitable sharing, contributing to better resource management and conservation within the population (Angourakis *et al*., 2015).

Individuals have the capacity to make decisions regarding cooperation, considering both the evolutionary equation and the social pressure of the group. This selection is influenced by the presence of cooperative and non-cooperative individuals (Hilbe *et al*., 2018). We suppose here that the behavior of the majority can condition others to adopt cooperation or non-cooperation options, thereby increasing the likelihood of individuals choosing the same strategy as those around them.

In addition, we have implemented into the model that individuals can decide to not cooperate at certain moments to address this limitation by incorporating additional factors that increase the possibility of non-cooperation decisions. In fact, humans in the simulation have the option to not cooperate based on three key assumptions: social pressure, the probability of non-cooperation (indicating a lack of interest or willingness to cooperate), and limited resources that implement competition between individuals. These factors influence the decision-making process and allow for variations in cooperative behavior throughout the simulation.

### Design of the Experiment

PaleoCOOP was used to perform simulations to test the impact of cooperation for the four scenarios. The selection of the factor values for the simulations can be listed in Table 4. Considering the aim of this work, the factors were chosen to first, explore the impact of the probability of the initial cooperation with different climate scenarios; second, examine the two factors alpha and cost of cooperation from the Cooperation model. Alpha will be tested to observe the importance of the degree of conformist transmission for cooperation when we change the environment. A lower value of alpha indicates that payoff-biased transmission dominates, while a higher value reflects stronger conformist tendencies, potentially leading to the persistence of common behaviors (Henrich & Boyd, 2001: 83). We also test the impact of cooperation costs by adjusting the level to observe how changes affect the overall benefit of cooperation.

**Table 4:** The factors explored in the model, including the probability of initial cooperation, the strength of conformist transmission (alpha), the cost of cooperation, and the climate scenarios. The probability of cooperation defines the likelihood of individuals initially adopting cooperative behavior. Alpha represents the degree of conformist bias in cultural transmission. The cost of cooperation determines the resources required for cooperative actions, impacting the overall viability of cooperation under different environmental constraints. The climate scenarios are designed to test the influence of varying temperature conditions

Factors	Range explored
prob-cooperation	[0.2 0.5 0.8]
alpha	[0.2 0.5 0.8]
cost-cooperation	[10 20 50]
scenarios	[“Scenario 1,” “Scenario 2,” “Scenario 3,” “Scenario 4”]

The factor ranges were selected to represent a plausible range of social behaviors in different environmental conditions. These values allow us to explore scenarios where these factors are rare, moderate, or prevalent on a different level, as well as the influence of varying levels of conformist transmission and cooperation costs on population survival.

Due to the extensive computational Requirements of this model, we segmented the simulations into individual scenarios, each spanning 1200 timesteps, with a total of 216 runs for all the scenarios. Furthermore, we employed multiple repetitions for each scenario within each simulation run to discern any potential variability within the sample during the simulation. Specifically, a number of three repetitions were conducted per scenario, and the results across these repetitions were consistent, showing minimal variation. While we agree that further repetitions could help quantify the internal variability more thoroughly, the computational constraints limited our ability to conduct additional runs. The consistency of the results, however, suggests that the model’s behavior was stable within the tested configurations.

## Results

### Probability of Cooperation

We assess the probability of cooperation in both the Tian Shan and Altai regions. Each scenario involves testing a different range of probabilities of cooperation (in a Range of 20%, 50%, and 80%) to explore how population dynamics evolve in response to varying degrees of cooperation. A complete list of figures for each simulation with all the parameters can be seen in the Supplementary Material.

#### Scenario 1: Average Annual Glacial-Interglacial Temperatures

**Fig. 4:**
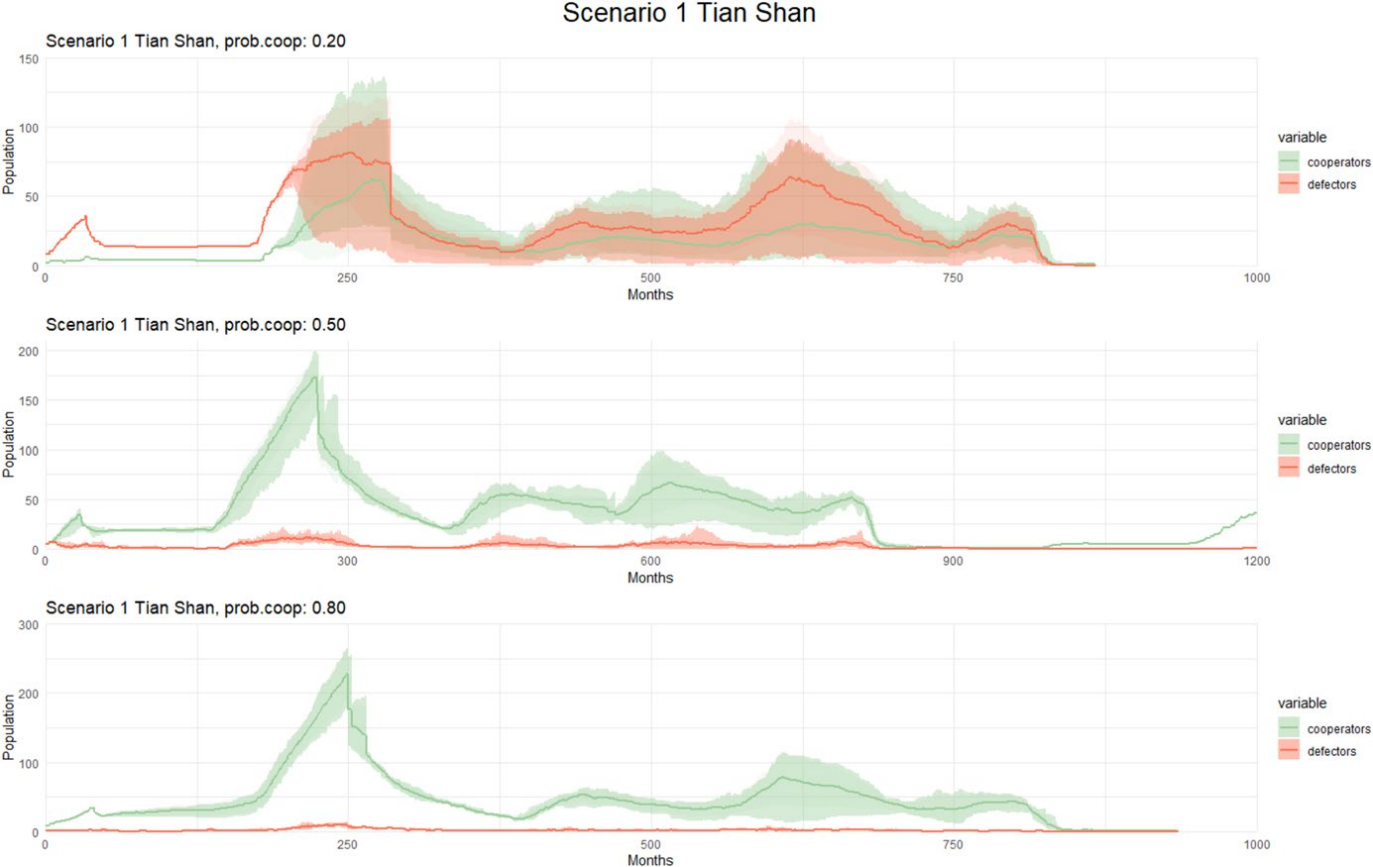
Population dynamics of cooperators and defectors over time (measured in months) in Scenario 1 (Tian Shan) with different cooperation probability. Each graph corresponds to a different cooperation probability, with varying levels of cooperation cost and the alpha parameter. Solid lines represent the mean population across simulations, while shaded areas indicate variability: the lighter band shows the full range (min–max), and the darker bands represent one standard deviation around the mean. These variations allow for a detailed analysis of how changes in these factors influence population trends and cooperative behaviors in the simulated environment

In the case of Tian Shan (see Fig. 4), we detect a significant increase in the number of cooperators when the probability of cooperation is equal to or greater than 50. Conversely, when the probability is Reduced to 20%, cooperators fail to increase in number within the population during the simulation, except when alpha is. Despite the increase of non-cooperators and defectors when the probability is set to 20%, individuals display lower chances of long-term survival and population increase compared to scenarios with a higher probability of cooperators.

**Fig. 5:**
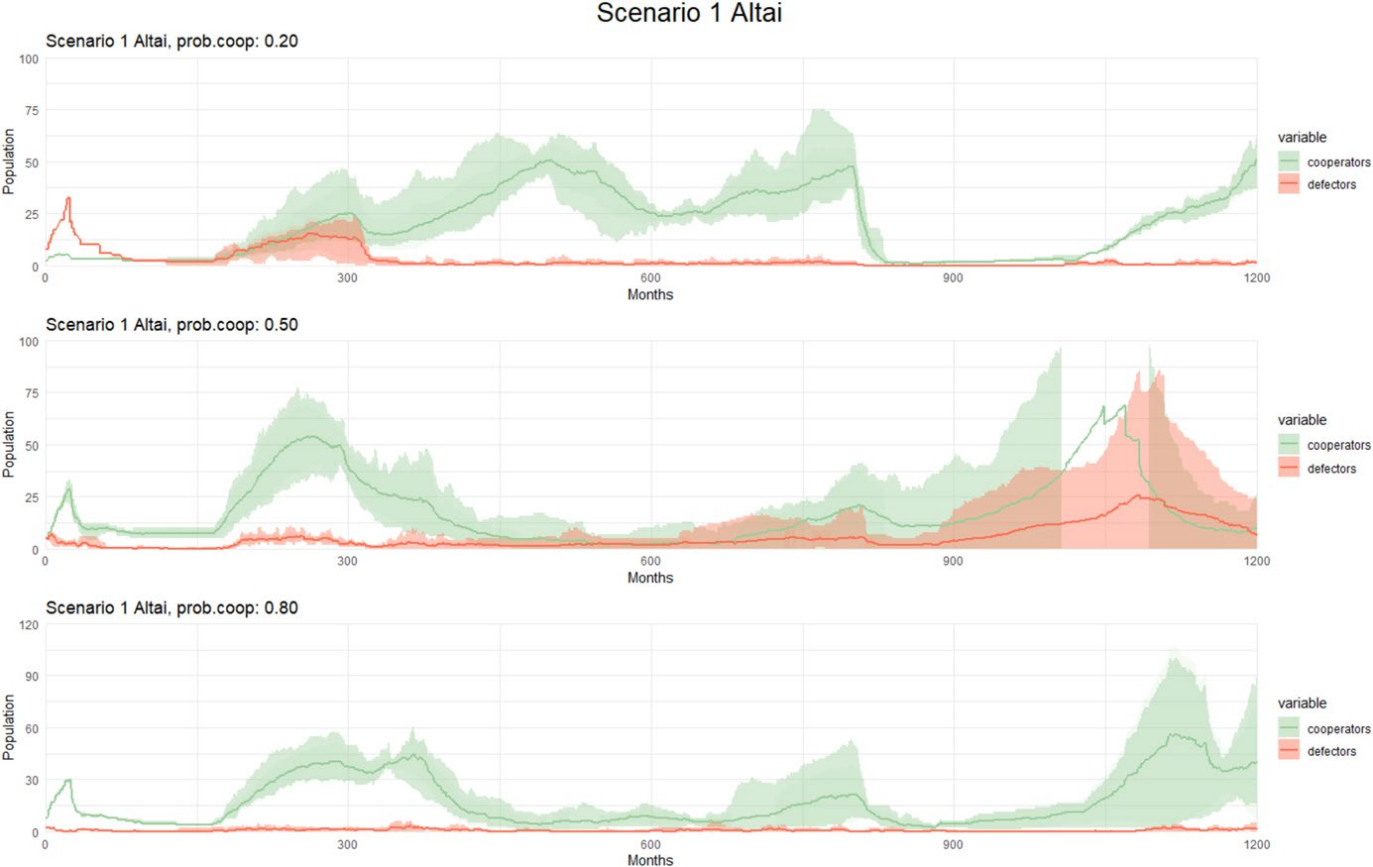
Observed patterns in Scenario 1 (Altai) with different cooperation probability. The graphs display population dynamics of cooperators and defectors over time (measured in months) for Scenario 1. Each graph corresponds to a different cooperation probability, with varying levels of cooperation cost and the alpha parameter. Solid lines represent the mean population across simulations, while shaded areas indicate variability: the lighter band shows the full range (min–max), and the darker bands represent one standard deviation around the mean. These variations allow for a detailed analysis of how changes in these factors influence population trends and cooperative behaviors in the simulated environment

In the Altai region**,** Scenario 1 displays a similar pattern comparable with fluctuations in population size attributed to varying climate temperatures (see Fig. 5). We observed minimal changes in population size and the presence of cooperators when the probability of cooperation is set to 20%, 50%, and 80%, with a slightly higher variation of the persistence of cooperators observed when the probability is 80%. As seen in Tian Shan, the scenario with a 20% probability of cooperation reveals a persistent presence of cooperators, increasing in numbers during the simulation.

#### Scenarios 2 (Glacial High Seasonality) and 3 (Interglacial High Seasonality)

We conducted separate simulations for Scenarios 2 and 3; however, we did not observe any discernible changes attributed to the minor differences in climate temperatures between glacial and interglacial periods in either the Altai or Tian Shan regions, as shown in Table 2.

**Fig. 6:**
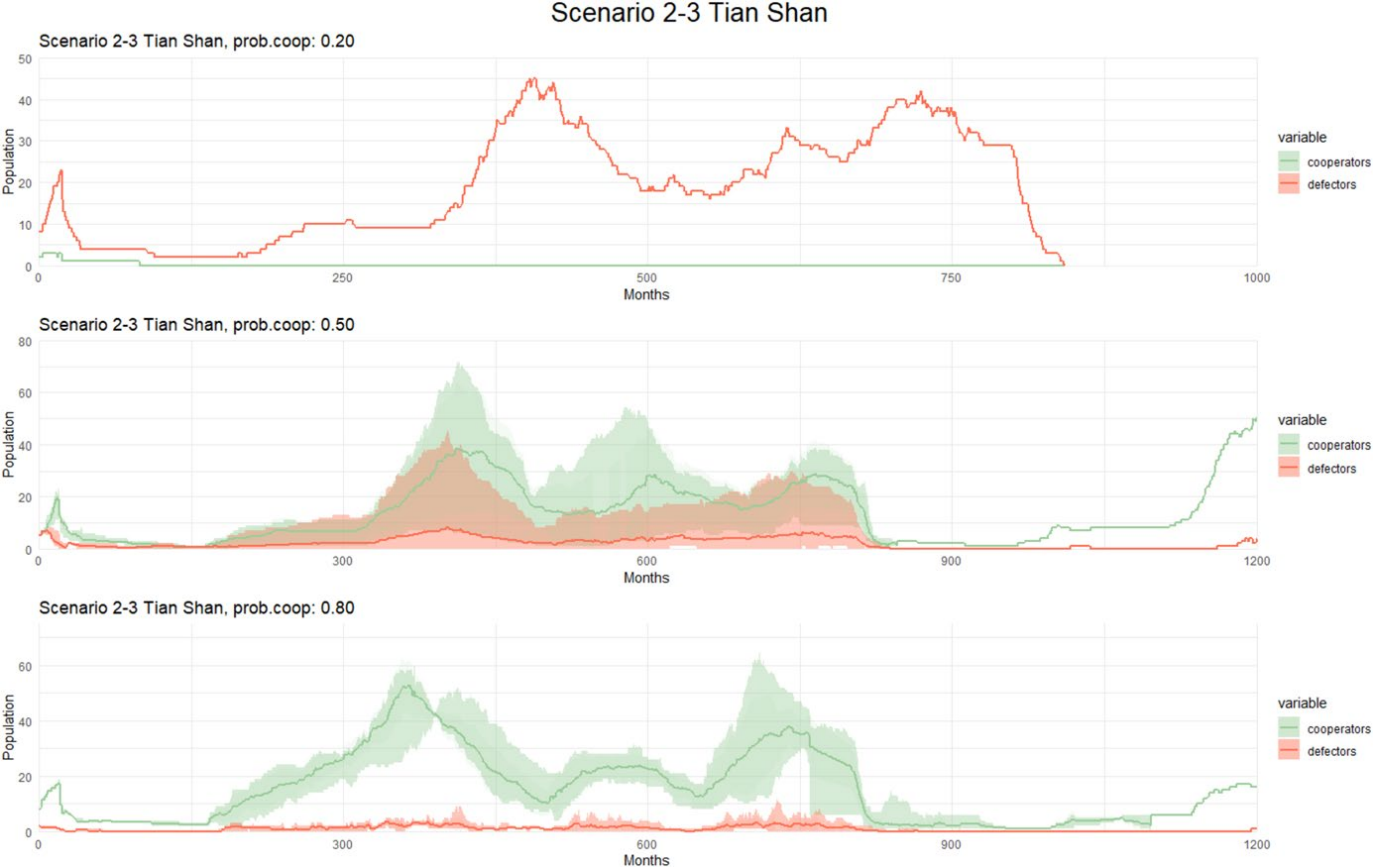
Population dynamics of cooperators and defectors over time (measured in months) in Scenarios 2–3 (Tian Shan) with different cooperation probability. Each graph corresponds to a different cooperation probability, with varying levels of cooperation cost and the alpha parameter. Solid lines represent the mean population across simulations, while shaded areas indicate variability: the lighter band shows the full range (min–max), and the darker bands represent one standard deviation around the mean. These variations allow for a detailed analysis of how changes in these factors influence population trends and cooperative behaviors in the simulated environment

**Fig. 7:**
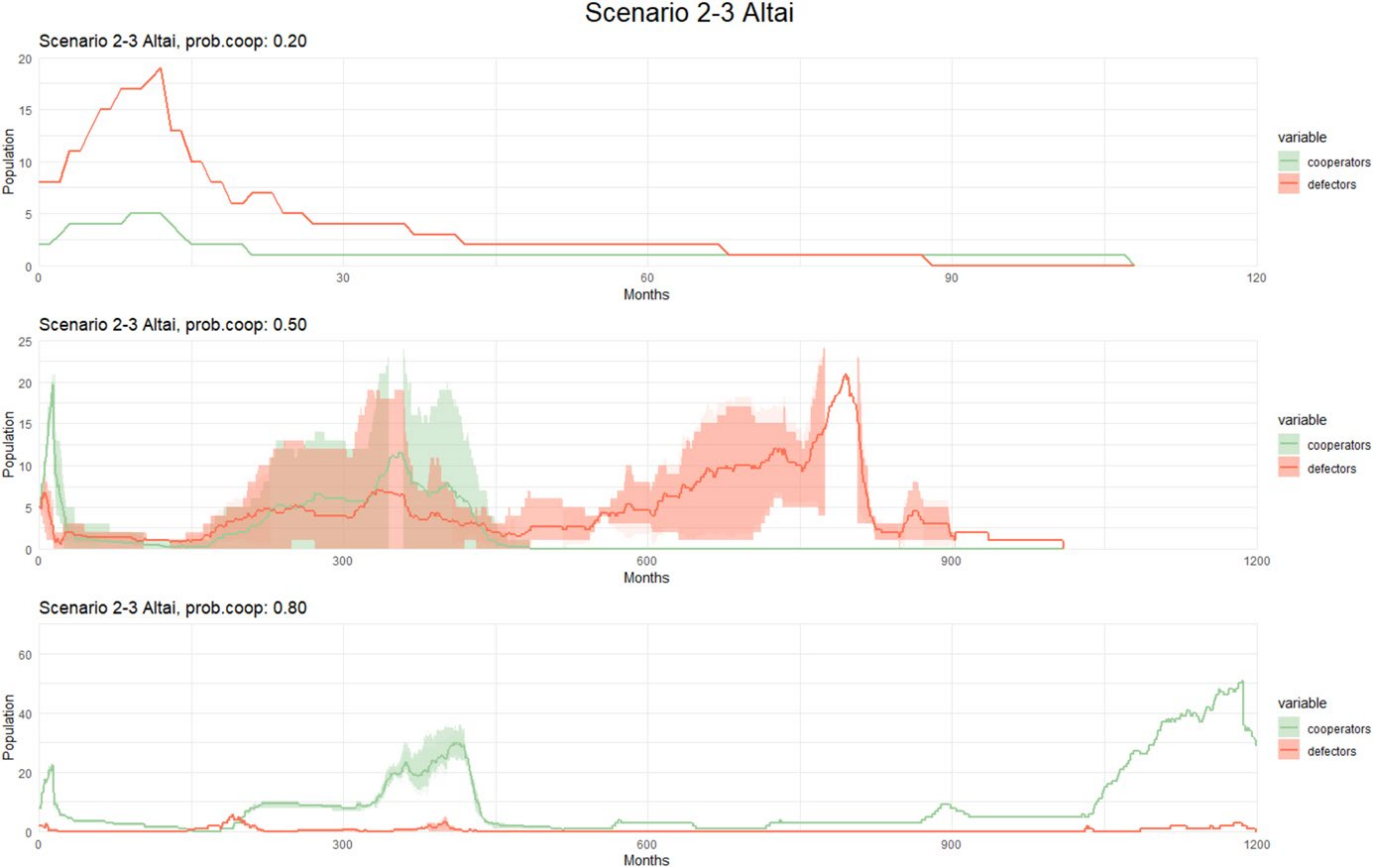
Observed patterns in Scenarios 2–3 (Altai) with different cooperation probability. The graphs display population dynamics of cooperators and defectors over time (measured in months). Each graph corresponds to a different cooperation probability, with varying levels of cooperation cost and the alpha parameter. Solid lines represent the mean population across simulations, while shaded areas indicate variability: the lighter band shows the full range (min–max), and the darker bands represent one standard deviation around the mean. These variations allow for a detailed analysis of how changes in these factors influence population trends and cooperative behaviors in the simulated environment

In both Tian Shan Scenarios 2 and 3 (see Fig. 6), we detect a slightly similar pattern emerging when the climate temperature becomes more extreme, especially when the probability of cooperation is below 50%. An increase in non-cooperators and defectors is observed but due to the extreme temperature in the case of the Altai, the total population does not increase (see Fig. 7).

In contrast, when the probability of cooperation exceeds 50%, the Altai and Tian Shan show different patterns in terms of survival. We observe a higher competition between cooperators and non-cooperators in the Altai than in the Tian Shan, especially when the cost of cooperation increases. Under these conditions, the probability of survival decreases in cooperators. However, cooperators tended to exhibit higher survival rates compared to non-cooperators throughout the simulation.

It is worth mentioning here that the climate temperature and environmental conditions in the Altai presented a higher level of hostility compared to the Tian Shan. Under these conditions, we identified a higher prevalence of survival when the probability of cooperation was set to 80% in Tian Shan. Surprisingly, the higher prevalence and the increase of population in Altai occurred when the cost of cooperation and alpha was lower.

The contrasting Results between the Tian Shan and Altai scenarios underscore the sensitivity of cooperative behaviors to varying environmental conditions and climate temperatures. In any case, in both the Altai and Tian Shan, the population grows less than in Scenarios 1 and 4.

#### Scenario 4: Warmest Interglacial

This scenario corresponds to a simulation involving the warmest temperatures in both the Altai and Tian Shan Regions, combining aspects of Scenarios 1 and 2, and specifically focusing on Resource consumption and energy usage. The aim was to test a potential warm scenario with Limited resources but a non-significant loss of energy compared to Scenarios 2 and 3.

In both regions (Tian Shan and Altai), we observed similar patterns in Scenario 4, mostly when the probability of cooperation is set to 80%. This could be attributed to a scenario where there are no significant differences in extreme temperature between them.

**Fig. 8:**
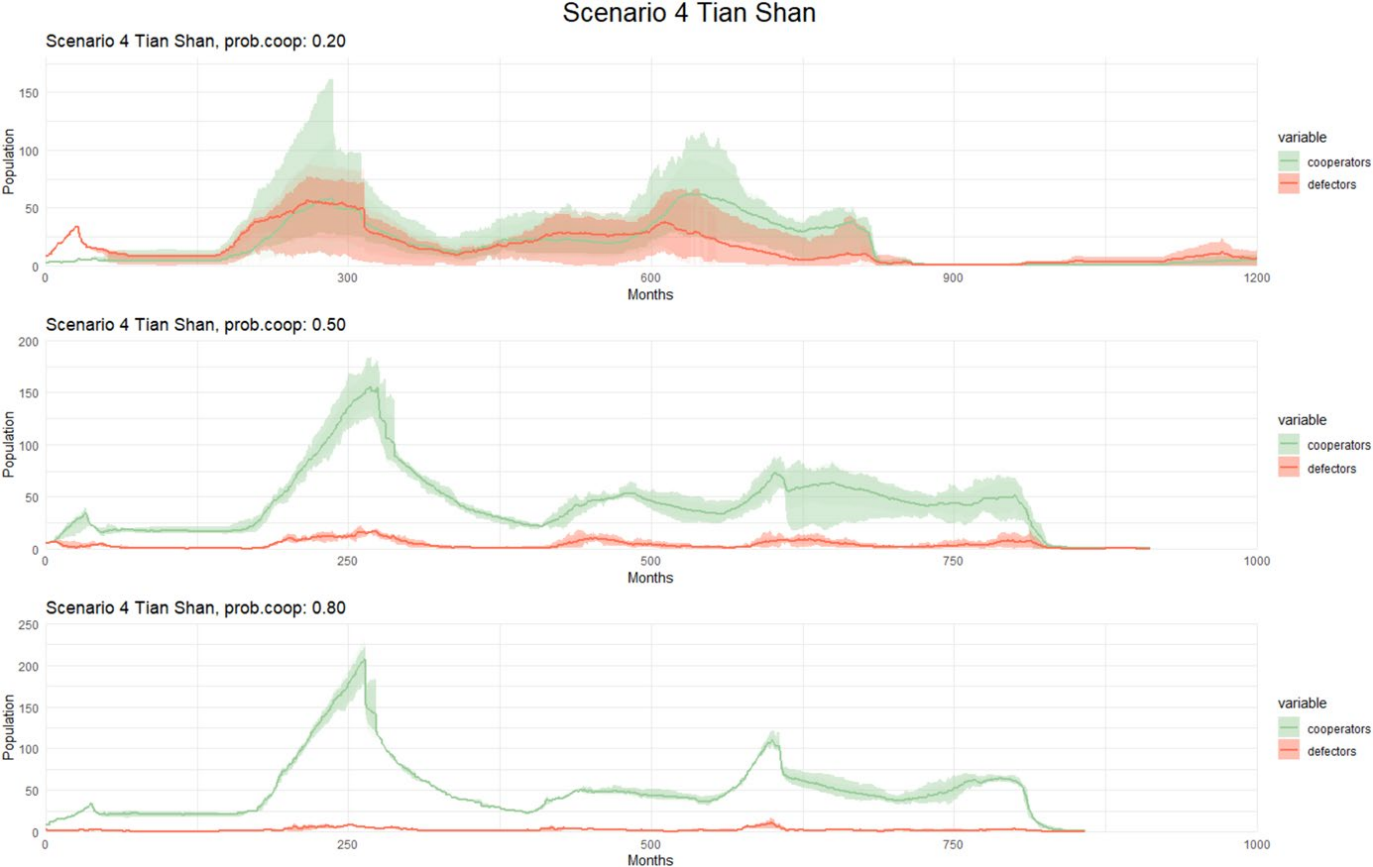
Population dynamics of cooperators and defectors over time (measured in months) in Scenario 4 (Tian Shan) with different cooperation probability. Each graph corresponds to a different cooperation probability, with varying levels of cooperation cost and the alpha parameter. Solid lines represent the mean population across simulations, while shaded areas indicate variability: the lighter band shows the full range (min–max), and the darker bands represent one standard deviation around the mean. These variations allow for a detailed analysis of how changes in these factors influence population trends and cooperative behaviors in the simulated environment

In the Tian Shan, Scenario 4 shows an increase in population size when the probability of cooperation was set to 50% and 80%, whereas the number of both cooperators and defectors is Relatively low when the probability is set to 20% (Fig. 8). Despite being a scenario without extreme temperatures, we can see cooperators struggling to maintain large populations when the probabilities are lower (20%) unlike Scenario 1 in the Tian Shan where cooperators outnumber non-cooperators by a majority.

**Fig. 9:**
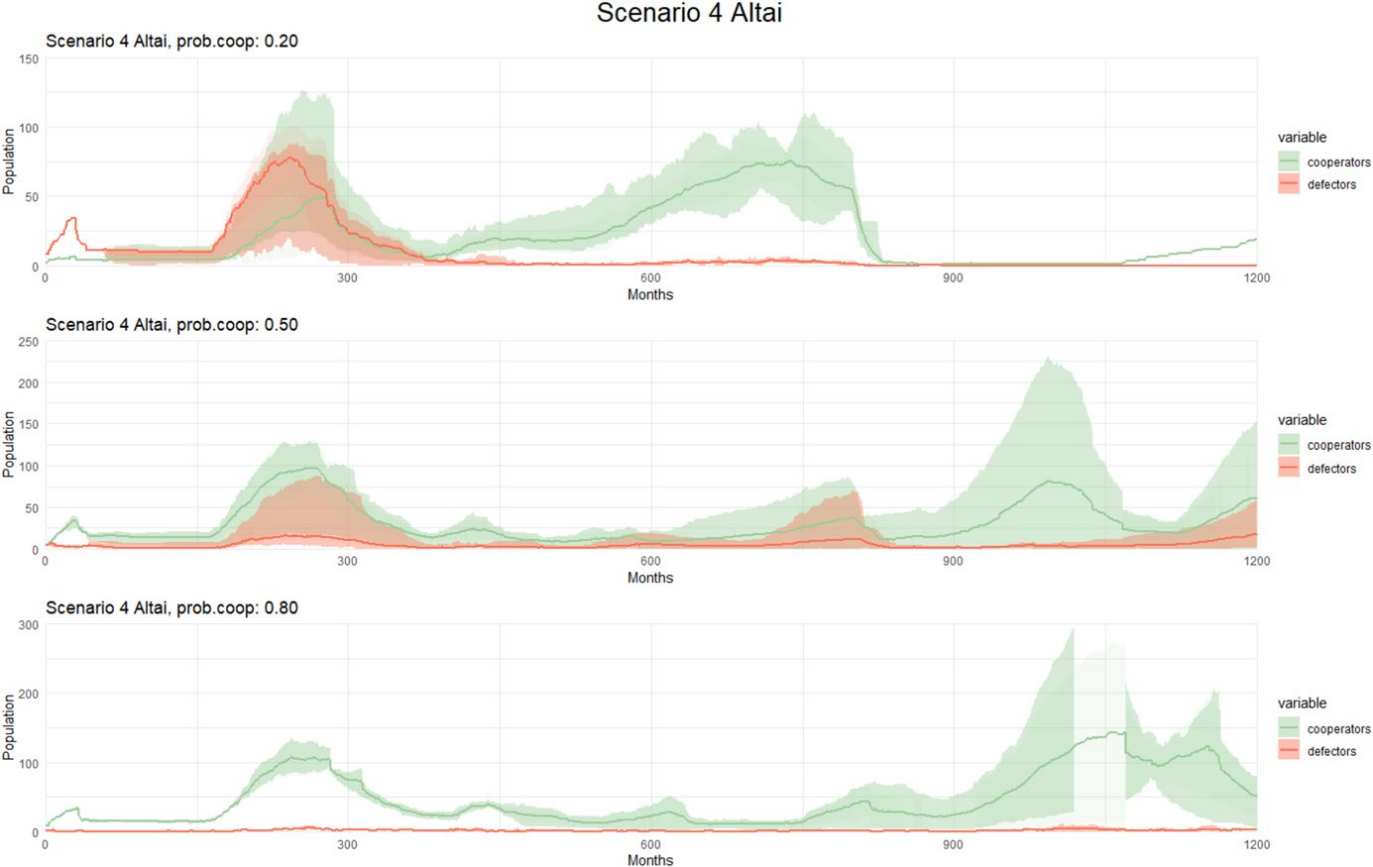
Observed patterns in Scenario 4 (Altai) with different cooperation probability. The graphs display population dynamics of cooperators and defectors over time (measured in months). Each graph corresponds to a different cooperation probability, with varying levels of cooperation cost and the alpha parameter. Solid lines represent the mean population across simulations, while shaded areas indicate variability: the lighter band shows the full range (min–max), and the darker bands represent one standard deviation around the mean. These variations allow for a detailed analysis of how changes in these factors influence population trends and cooperative behaviors in the simulated environment

When the probability of cooperation was set to 80%, the Altai followed a similar pattern to the Tian Shan, albeit with a higher increase in population size (Fig. 9). When the probability of cooperation was 20%, the survival Rate of cooperators in the Altai was higher than in the Tian Shan, but they hardly differed when the probability of cooperation was set to 50% in both regions, except when the alpha is higher in the Altai Scenario.

### The Influence of Alpha and the Cost of Cooperation in the Scenarios

After analyzing the initial probability of cooperation within the model, we focus on the impact of alpha and the cost of cooperation. Alpha, representing the strength of conformist transmission, delineates an individual’s inclination to imitate prevalent actions within their society, while the cost of cooperation defines the expense borne by each cooperator, which resonates uniformly within the group. Non-cooperation evades this cost while benefiting equally.

The goal of conducting these simulations was first, to explore the model’s sensitivity across varying parameters and its substantial influence on the results; second, to evaluate the effects of both the conformist transmission value and the cost of cooperation within the model; and finally, to determine the potential effect or interaction between alpha and the cost of cooperation on the overall dynamics within the model.

Our simulation results indicated significant changes with increasing values of alpha and the cost of cooperation. Importantly, we observed that variations in the initial probability of cooperation had a more pronounced impact on the model results than adjustments to alpha and the cost of cooperation.

This especially affects scenarios where the probability of cooperation is set to 20% with the increase of defectors when the alpha and cost of cooperation are higher. Across all scenarios, increasing the cost of cooperation reduces the population of cooperators and increases the relative proportion of non-cooperators and defectors, but the effect is less pronounced when the probability of cooperation is high (80%).

On the other hand, in Altai and Tian Shan, increasing alpha is not perceived as having a significant effect when the probability of cooperation is 80% in non-extreme scenarios. Across all scenarios, increasing the cost of cooperation reduces the cooperator population and increases the relative proportion of defectors, but the effect is less pronounced when the probability of cooperation is high (80%). However, we can detect a different pattern in more extreme scenarios (Scenarios 2–3) when the level of alpha and the cost of cooperation are higher with lower population growth and higher competitiveness.

In Scenario 1, population dynamics in the Tian Shan and Altai regions are largely affected by the probability of cooperation. Higher cooperation (50% or above) leads to a more stable population, whereas lower probabilities (20%) see fewer cooperators and less population growth, especially in extreme climates. Scenarios 2 and 3 show a weaker impact from temperature fluctuations between glacial and interglacial periods, but cooperation Remains vital for survival. Scenario 4, the warmest period, shows similar trends, with higher cooperation correlating to better population outcomes. Alpha and the cost of cooperation further influence these dynamics, especially under extreme conditions.

## Discussion

### Climate Scenarios and Cooperation

Our results suggest that climate change can have a substantial impact on cooperative tendencies in human populations, with higher probabilities of survival observed when cooperation levels are elevated.

The model also indicates that the initial probability of cooperation plays a significant Role in shaping group behavior, whether it is for the benefit of the cooperators or the defectors. This highlights the influence of individual preferences and predispositions on the overall cooperation dynamics within a population. We also detect differences in population size between the regions when temperatures are colder. More specifically, populations decrease in scenarios where winter temperatures are extremely low and increase in periods where the coldest quarter is milder in Scenarios 2–3 (glacial-interglacial high seasonality).

In Scenarios 1 and 4, the share of cooperators increases when the probability of cooperation is set to 80%, and this pattern holds for both the Tian Shan and the Altai. In Scenarios 2 and 3, this increase is not as pronounced. These findings emphasize the pivotal Role that the probability of cooperation plays in shaping cooperation patterns and the resilience of human populations. At 80%, this higher probability of cooperation overwhelms all other factors that could negatively influence cooperative behavior (*e.g.*, the cost of cooperation), leading to the cooperators’ dominance and promoting their survival in all climate scenarios. Our model demonstrates that larger populations could imply a higher pressure to cooperate. This aligns with previous research highlighting the role of group dynamics and social pressures in promoting cooperative behaviors (Aktipis *et al*., 2016; Cornwallis *et al*., 2017; De Jaegher, 2017; Liu & Chen, 2018; Martin *et al*., 2020; Pereda *et al*., 2017).

In Scenarios 2 and 3, a high probability of cooperation has a positive impact on survival. Specifically, in the Tian Shan Region, the number of cooperators increases to the exclusion of non-cooperators who disappeared before the simulation concluded. A large number of cooperators pushes non-cooperators to switch their strategy, leading to an increase in the number of cooperators. Indeed, this does not occur when the probability of cooperation is lower than 50%. Some empirical data supports the notion that, in situations of high pressure and scarce resources, a highly cooperative strategy may be more viable than other individual survival mechanisms (Lange *et al*., 2017; Testard *et al*., 2024). However, with a lower probability of cooperation, other more individualistic strategies may emerge. The shift toward individualistic strategies often occurs as a means of self-preservation. Where competition for limited resources becomes intense, individuals tend to focus on individualistic rather than cooperative strategies. The balance between cooperative and individualistic strategies can vary depending on various factors such as the proximity of attractor places and non-optimal resources.

This situation is not visible in Altai, where temperatures are even more extreme than in Tian Shan. We observed that a strategy with a probability of cooperation lower than 80% appears to be ineffective in extremely cold conditions. We noticed a striking trend where a lower probability of cooperation consistently Resulted in a population decrease to zero. Scenarios 2 and 3 in the Altai suggest that the impact of the probability of cooperation on survival is influenced by the specific environmental context (Altai vs. Tian Shan), the proximity of attractor places to each other, and the availability of resources.

As observed in the coldest scenarios, reducing population size may prove adaptive for surviving extreme climates. This strategy appears to be an effective response to harsh conditions, as it can improve the chances of resource availability by having cooperators save resources, thus increasing the probability of survival in these extreme scenarios.

Furthermore, our model demonstrates that the initial probability of non-cooperation can strongly influence cooperative dynamics within human groups. Thus, if the initial probability of non-cooperation is higher, then groups will tend to defect even if a cooperative subgroup pushes them to cooperate. In extreme scenarios (Scenarios 2–3), if the initial probability of cooperation is set to the lowest level (20%), cooperative subgroups may not even emerge or, if they do, they may not be sufficient to drive overall group cooperation.

### Cost of Cooperation and Alpha

Our simulations reveal that the initial probability of cooperation has a stronger influence on population dynamics than changes in alpha (strength of conformist transmission) or the cost of cooperation. While increasing the cost of cooperation reduces the number of cooperators and increases defectors (strategy-switchers), this effect is less pronounced when the cooperation probability is high (80%). In contrast, when the cooperation probability is low (20%), the influence of high alpha and cooperation cost values is significant. Specifically, more non-cooperators and defectors emerge under these conditions and the population is less stable.

Alpha has a limited impact in non-extreme scenarios but becomes more significant in harsher environments (Scenarios 2 and 3 in both regions). In these scenarios, higher alpha and cooperation costs result in populations decreasing and more competition. Interestingly, this suggests that fostering cooperation from the beginning is crucial and that alternative mechanisms beyond alpha and cost may be needed to sustain cooperation under extreme conditions.

An increase in alpha (the strength of conformist transmission) with a low initial probability of cooperation suggests that when people are more influenced by what the majority does, they tend to adopt non-cooperative behaviors. In contexts where cooperation is rare (low probability), people may be more inclined to follow the behavior of the majority. This may occur because, by following the majority, people choose to defect rather than cooperate, especially if defection is more common in the group. On the other hand, a decrease in alpha leading to an increase in cooperators indicates that people are more willing to evaluate the benefits of cooperating. This means that in situations where cooperation is advantageous, people tend to adopt those behaviors, as they are not as influenced by the prevailing social norm.

### Cooperation vs. Resources

Resource optimization also seems to be a key factor influencing cooperative behavior. We observed different decision-making strategies in the competition for resources between cooperative and non-cooperative individuals. Non-cooperators tended to monopolize resources, which became suboptimal, leading to faster migration to another place. In contrast, cooperators exhibited a higher likelihood of survival, which we attribute to their effective resource utilization and their willingness to share resources. This behavior proves advantageous during periods of environmental change.

We also observed a tendency for populations to decrease in size during significant migrations in the search for new optimal Resources. These temporary fluctuations in the population could signal a survival strategy for cold temperatures because smaller populations may use resources more efficiently. However, when the probability of cooperation falls below 50%, this pattern changes. In such instances, non-cooperators initially proliferated but subsequently declined. This can be linked to their exploitative strategies driven by the initial abundance of resources when non-cooperators over-consume based on initially abundant resources. However, as competition intensifies, this strategy becomes detrimental, and their numbers subsequently decline.

An additional critical factor influencing the cooperation strategy involves the proximity of available resources and attractor places to each other (their density). This is particularly crucial in more extreme scenarios. The initial stages of searching for better resources and the resulting relocation could cause fluctuations in population size as agents adjust their strategies. Specifically, there are short-term spikes in population size before the system stabilizes at a new location. During these fluctuations, agents might shift their cooperation strategy more frequently as they experiment in the new environment. After migration, cooperation might temporarily flourish, leading to peaks in the cooperator population. However, as resources decrease again, the population might stabilize or decline, leading to a subsequent drop in population after the peak.

On the contrary, the cooperation strategies do not produce the same effect in non-extreme scenarios (*e.g.*, scenarios 1 and 4). We detect significant changes in cooperation strategies when the distance between groups of individuals and resources increases. This also happens when attractor points that provide protection from the cold are farther from or closer to each other. This observation suggests a connection in the model between cooperation strategies and the availability of resources and shelter. Therefore, cooperative strategies have a direct impact on long-term survival and stability, especially in resource-scarce environments.

One limitation of our approach is that resource-rich areas (attractors) were predefined based on archaeological data, which may influence cooperation dynamics. However, since agents can utilize resources beyond these attractors, the model does not assume that survival is only possible in these locations. Future studies could explore alternative resource distributions, including more widely dispersed but lower-yield resources, to assess how different ecological conditions influence cooperative behavior.

## Conclusion

The findings of our simulation model shed light on the intricate relationship among climate, cooperation behavior, and survival in extreme conditions. In this study, we present an evolutionary framework to examine the role of cooperation in human mobility within the context of climate change. Our objectives were to investigate if cooperation strategy can be essential for survival in extreme climate conditions by selecting one strategy.

We show that a high probability of cooperation can increase the chances of survival in the hardest conditions in both regions. In the Altai, where the coldest temperatures are modeled, this effect is somewhat reduced even when cooperation is high.

These results may contribute to our understanding of why Central Asia may have been settled later by modern humans compared to other regions in Asia, shedding light on the importance of behavioral adaptations in human dispersals under adverse conditions.

We also found that the distribution of resources has an impact on cooperative behavior. Cooperators demonstrate more effective resource utilization and resource sharing, proving that cooperation is advantageous during periods of scarcity.

Further, population reduction emerges as an effective survival strategy response to extreme climates, enhancing the efficiency of resource utilization and increasing the likelihood of survival. This adaptation attempt might have crucial implications for understanding human responses to hostile environments.

Computational models can provide a fundamental baseline for future research on dispersal and behavioral adaptations in ancient societies. By simulating and exploring diverse behavioral scenarios, these models offer a powerful tool to investigate the complexities of the past.

The insights gained from such studies contribute to a broader understanding of Central Asian prehistory and provide a foundation for future studies on cooperation and human adaptation in the face of environmental challenges.

## Supplementary Information

Below is the link to the electronic supplementary material.

Supplementary file 1 (ZIP 2.34 MB)

Supplementary file 2 (PDF 373 KB)

Supplementary file 3 (ZIP 18.1 MB)

Supplementary file 4 (ZIP 2.33 KB)

Supplementary file 5 (PDF 2.12 KB)

## Data Availability

Code, data and sources are available at Github archive is openly available under Open licenses here https://github.com/Mcotsar/PaleoCOOP and OSF repository 10.17605/OSF.IO/JM3ZY.

## Declarations

### Open Data

PaleoCOOP Model was written on NetLogo software 6.2.2 (Wilensky & Rand, 2015) and the code, data and sources are openly available at Github archive under Open licenses here https://github.com/Mcotsar/PaleoCOOP. As part of open science and transparency policies in archaeology, the agent-based model was performed according to practices following the ODD document protocol (Grimm *et al*., 2010, 2020; Müller *et al*., 2013; Polhill *et al*., 2008). Code, data, sources and supplementary information on the model can also be found here 10.17605/OSF.IO/JM3ZY. Data have been analyzed and conducted in R program version 3.2.4, using the packages *dplyr* (Wickham *et al*., 2019), *ggplot2* (Wickham *et al*., 2016) and *reshape2* (Wickham, 2020). Maps were performed using QGIS software v.3.28.4-Firenze, POSITRON ASTER Global Digital Elevation Model V003.
