# Supplementary material for: The Impact of Cooperation Under Climate Constraints: An Agent-Based Model for Exploring Paleolithic Behavioral Adaptations in the Inner Asian Mountain Corridor
Source: J Archaeol Method Theory. 2025 Sep 20;33(1):8. doi: 10.1007/s10816-025-09739-z (PMC12450231; doi:10.1007/s10816-025-09739-z)
Supplement: Supplementary file 2 — (PDF 373 KB) [file 10816_2025_9739_MOESM2_ESM.pdf]

PaleoCOOP model

Authors: María Coto-Sarmiento, Abay Namen, Aristeidis Varis, Radu Iovita

Model designed by: María Coto-Sarmiento

Protocol for the preprint:

Coto-Sarmiento, M.; Namen, A.; Varis, A. and Iovita, R. (submitted 2025): "The impact of cooperation under climate constraints: an Agent-Based Model for exploring Paleolithic behavioral adaptations in the Inner Asian Mountain Corridor".

Introduction and Open Data

PaleoCOOP Model was written on NetLogo software 6.2.2 [1] and the code, data and sources available at the Github archive are openly available here https://github.com/Mcotsar/PaleoCOOP.

As part of open science and transparency policies in archaeology, the agent-based model was performed according to practices following the ODD document protocol [2] [3] [4] [5]. Supplementary information on the model can also be found here: https://osf.io/jm3zy/

Overview

Purpose and Patterns

PaleoCOOP is an Agent-Based Model that simulates human cooperation against climate constraints with an evolutionary framework. This model primarily focuses on exploring the impact of behavioural adaptation on the survival of groups of individuals in extreme climate environments.

PaleoCOOP provides insights into cooperative behaviour, resource competition, and the ability to thrive in challenging climatic conditions by analyzing the interplay between human decision-making and behavioural adaptation in extreme conditions:

a) Human cooperation: the model tests the influence of extreme environments on human cooperation.
b) The adaptation mechanism focuses on how individuals learn from one another and adjust their behaviours to promote group survival.
c) The mechanism for adaptation is to understand how individuals learn from one another and adapt their behaviours to promote group survival.
d) Competition for optimal resources: Competition for access to better resources can shape individual behaviour. This study explores the relationship between resource availability, cooperation strategies, and group competitiveness.
e) Survival in extreme climatic environments: The model examines how groups of individuals adapt and survive under different climatic conditions.

PaleoCOOP explores four theoretical climate scenarios, each representing a climate environmental condition, from less extreme to more extreme. These scenarios serve as theoretical settings to analyze and compare the adaptive strategies for human cooperation. By examining the effects of climate variation on cooperative behaviour, the model enables a comprehensive exploration of the different factors that contribute to individuals adopting cooperation or defection in different climatic contexts.

Entities, state variables and scales

Environment

The model uses a GIS package on NetLogo and requires an ASCII elevation raster map. The maps also use shapefiles with original sites and lakes and rivers. The map

of the environment is represented by a grid of patches (534 x 328, see the article for a better description). Water and mountains are represented with a shapefile and ASCII elevation raster shapefile, respectively. We created two environments for the model: Altai and Tian Shan, divided into two geographical spaces, Altai and Tian Shan within the territory of modern-day Kazakhstan. Each patch on the map represents 0.5 km² of the geographical area (1 patch size in pixels).

The factors of the environment are represented in **Table 1**.

Name	Overview Description
`basemap-dataset`	Map used with QGIS extension NetLogo
`attractors-dataset`	shapefile that includes archaeological data
`river-dataset`	shapefile that includes rivers
`lakes-dataset`	shapefile that includes lakes
`display-attractors`	Button. Display the archaeological sites located in Tian Shan and Altai
`clean-maps`	Button. Clean all and reset the shapefiles
`elevation`	Convert the elevation of a DEM.asc map to the model
`min-elevation`	Minimum elevation of the DEM (m)
`max-elevation`	Maximum elevation of the DEM (m)
`patches-region-tienshan`	Patches are divided into two regions: Altai and Tian Shan. This corresponds to patches of the region of Tian Shan
`patches-region-altai`	Patches are divided into two regions: Altai and Tian Shan. This corresponds to patches of the region Altai
`climate-temperature`	Patches are divided into two environments that include different climate temperatures in the Tian Shan and Altai. The temperature also depends on the climate scenarios selected
`resources`	Resources on the patches and attractors

	places. Consumption of resources will depend on the different scenarios. Default level: 50 (random area), 100 (attractive area)
`noresources?`	Boolean. True: resources are less than 20 % optimal on the patch, and False: there are enough resources on the patch (more than 20 % optimal)
`resource-decrease-rate`	Resources are decreasing at every time step. Default level: 0.1
`resource-recovery-rate`	Resources start to recover after they are not optimal at every time step. Default level: 1
`attractors?`	Boolean. True: identify patches with potential attractor places. False: otherwise
`origin-tienshan?`	Boolean. True: patches from Tian Shan, false: patches from Altai
`origin-altai?`	Boolean. True: patches from Tian Shan, false: patches from Altai.
`scenario`	Chooser. Selection of scenarios.

Table 1. Environment Factors. Includes also buttons.

Agents

The model incorporates three different entities (agents): groups of humans from Tian Shan and Altai, and attractors places. The two groups of humans are initially located in different random locations within the Tian Shan and the Altai. The specific characteristics of humans from each area are outlined in **Table 2.** To ensure a more homogeneous model design, both groups of humans possess identical features at the beginning of the simulation.

The parameters for the agents (human agents and attractor places) are described in **Table 2**.

*** NOTE:** In the model code, hominins and heminins have the same features in the model. Hominins correspond to human agents from Tian Shan and heminins represent human agents from Altai. We use different terms to differentiate for simulation.

Name	Overview Description
`places`	Attractor place agents where humans go for resources and protection from cold temperatures. They have different colors: red from Tian Shan and blue from Altai. The attractors represent sites with greater resource availability. These attractors are randomly placed within the geographical environment where the high availability of resources is located. The attractor's places are independent of patches with more resources and they possess their own set of resources
`attractor-resources`	Extra resources from attractor places. Default level: 100
`hominins`	Human agents from Tian Shan.
`heminins`	Human agents from Altai.
`Nhominins`	Define the number of individuals (from Tian Shan and Altai) in the simulation.
`color`	Colors of the humans to distinguish their behavior on the model. Greens are cooperators and Reds are defectors in the model. Blacks are humans under 12 years old; they have not decided yet.
`age`	Age of the humans. Default level: random 0-50
`maximum-age`	The maximum age of humans. Average 50 years old.
`minimum-reproduction-age`	The minimum reproduction age of humans. The reproduction rate is 12 years old.
`learning-rate`	Define the probability of learning the path to reach a destination. It depends on the difficulty

	determined by the climate. The default level is 1
traits	Define how hominins transmit and spread traits to others. Hominins acquire a trait when they are 12 years old. The default level is 1 for cooperation and 0 for non-cooperation. Note: this factor is not included in the model results
energy	Energy of humans. 0 = no energy 100 = entire energy.
max-energy	The maximum energy that humans can start the simulation. Default level: 100
risk	Risk of humans related to human risk
human-risk	Risk that human agents perceive. Risk is related to facing difficult situations to survive. Default level: 5
body-temperature	Body temperature of human agents
initial-body-temperature	Initial body temperature of human agents. The initial body temperature is 37
hominin-speed	Speed of the human agents on the landscape. Default level: 0.75

Table 2. Agent factors (Human agents and attractor place agents)

Process Overview and Scheduling

We simulate four theoretical scenarios, with each time step representing one year (one tick = one month). The simulation spans 1200 time steps (months), the equivalent of 100 years.

The model develops distinct stages throughout the simulation, which can be observed in **Fig. 1**. These stages include learning, interaction (cooperation model), resource consumption, and ultimately, moving to another location.

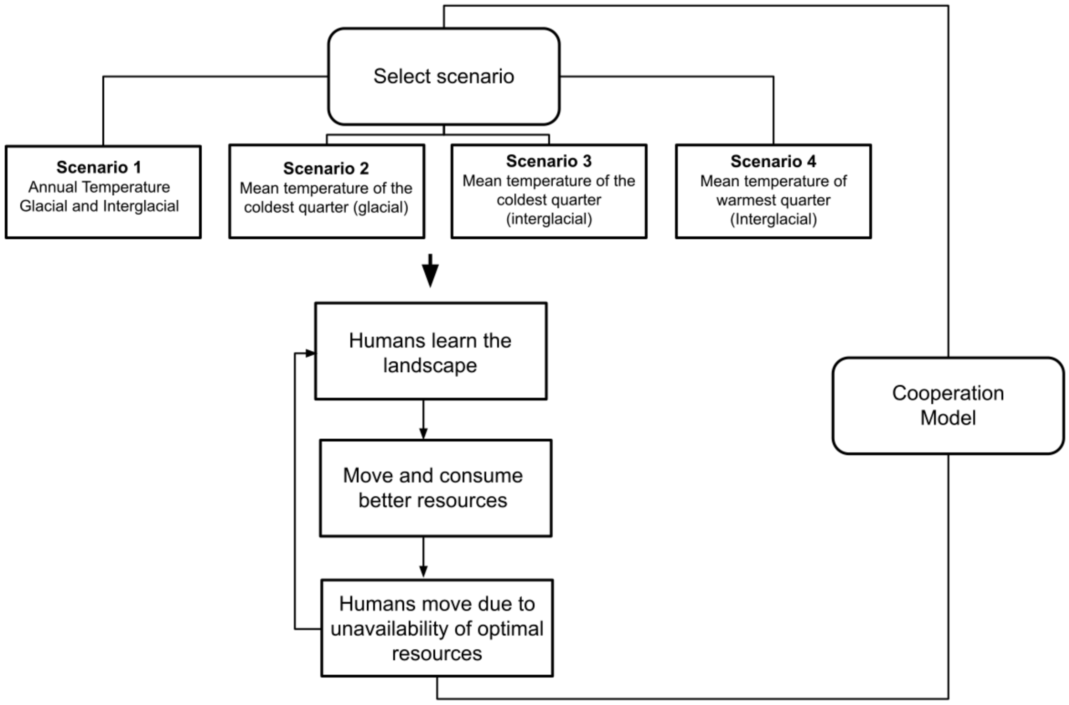

Fig 1. A diagram of the stages for the model.

At the beginning of the simulation, humans can move through the landscape while simultaneously acquiring the knowledge required to identify areas with better resources. Once individuals learn the optimal path to reach a place, they move toward areas for foraging. Upon migrating to a new location, the first stage of the model starts again in the new place, continuing until resources become limited within that area. At such a point, individuals are once again compelled to move, either back to their original place of origin or to alternative locations in proximity.

Design Concepts

Basic principles

PaleoCOOP uses an evolutionary agent-based model to test four theoretical climate scenarios under cooperation dilemmas. The model addressed significant questions, such as how humans survive under harsh conditions, the role of social strategies under different climate temperatures, and whether these strategies enhance human adaptability in extreme conditions.

The fundamental principles for the PaleoCOOP model are a) climate change can influence human cooperation, b) a larger population can be influenced by the pressure of the group to deal with cooperation dilemmas, and c) the selection of one strategy is contingent upon the percentage of cooperation observed in the model.

We provided insight into our model by testing four different theoretical climate scenarios in order to analyze how the survival of humans can be influenced by the selection of a specific strategy.

Adaptation

The model indirectly reflects adaptation by simulating diverse climate scenarios, requiring human agents to adjust and acclimatize to their respective climate environments (for more information, refer to the *Learning and Cooperation subsection*).

Objectives

This model simulates groups of individuals aiming to enhance their behavior by learning and adapting to their climate environment. Their goal is to discover superior

resources while engaging in competitive interactions. Additionally, they endeavour to optimize foraging by identifying attractive resting spots to replenish energy and regulate body temperature.

Learning and Cooperation

The PaleoCOOP model includes learning by allowing agents to acquire knowledge about the path to find optimal resource acquisition. The decision rules in the model can evolve during the simulation, particularly within the cooperation model.

At the beginning of the simulation, humans engage in a learning process and individuals must learn how to identify attractors and find them, which correspond to locations abundant in resources.

The cooperation strategy plays a significant role in this model as humans can select a social strategy between cooperation and defection over time (refer to the Cooperation submodel subsection). The strategy can be modified during the simulation based on various factors, such as a) the cooperation model, b) social pressure, and c) competition for limited resources which implements the competition among individuals to pursue better alternatives.

Mobility patterns

The model includes the analysis of mobility strategy patterns and resource availability [5] [8]. We previously defined a place of origin where hominins initially arrive, and a secondary location to which humans later migrate (for further information, refer to the article).

Sensing

At the beginning of the simulation, all agents are initially unaware of their environment. Thus, they must learn how to become familiar with their environment and identify the best leading to high resources. Once the path is learned, individuals become capable of detecting better resources within the set radius of perception and directly heading toward them. The optimal resources are located in attractor locations with potential shelter for humans. Here we introduce the concept of perceptual learning, whereby agents actively explore and perceive their surroundings to optimize their movements, seeking resources while dealing with the climate. Upon moving once more, human agents need to relearn the new environment.

Interaction

Interaction occurs through the cooperation model. The adoption of a strategy will depend on the interaction of the human agents with others (see *Cooperation subsection* for more details).

Stochasticity

Although this model is not stochastic *per se*, stochasticity is used in various aspects concerning the human agents and environments within the model.
First, during the initialization, the distribution of patch values is influenced by the dataset of observed places. Based on these data, the environment is reconstructed in a way that locates patches with higher resource abundance closer to these archaeological places of interest. For the remaining environments, the distribution of resource values is stochastic *in nature*.
Second, the initial positions of the human agents in Altai and Tian Shan, as well as their initial movements, incorporate a level of randomness used in the `learning-rate`. However, once agents locate resources with higher value, they

proceed directly toward these resources, while subsequent movements remain random. In addition, patterns of random movement in the model were included for the humans. Third, human agents possess the capability to create new attractor places. The new attractor places are randomly distributed in the environment.
This introduces additional stochasticity to the model, allowing for exploration and variability in the placement of attractor locations.

Collectives

Social structure is not specifically included in the model, a group of individuals is moving but each agent represents one individual with one social behavior.
In contrast, collectives are included in the cooperation model through social pressure by using a `radius-for-strategy` in the model (see *Cooperation Model section*).

Observation

The observation outputs of the model can be seen in **Table 3.**

Factor	Overview Description
`population (count hominins/heminins)`	Count the total number of human agents
`cooperate? = true`	Count the total number of human agents that cooperate
`cooperate? = false`	Count the total number of human agents that do not cooperate/defect
`found-newplaces-tienshan` `found-newplaces-altai`	Count the total of the attractor places created in Tian Shan or Altai
`payoff-tienshan` `payoff-altai`	Calculate the payoff. See the *Cooperation model section*
`delta-tienshan` `delta-altai`	Calculate the delta. See the *Cooperation model section*

`death-hypothermia`	The total number of human agents who died from hypothermia. Not included in the model results
`death-old`	The cumulative death toll attributed to natural causes or old age. Not included in the model results
`death-starvation`	The total number of human agents who died from starvation. Not included in the model results

Table 3. Observation output factors

Initialization

Environment initialization (map): the model consists of a theoretical model environment divided by 534 x 328 patches. Each patch on the map represents 0.5 km² of the geographical area (1 patch size in pixels). We use a map for the model generated using the Geographical Information System (GIS) package on NetLogo. We added to the model shapefiles of rivers, lakes and archaeological data. In the model, we use a shapefile of points of the dataset of potential archaeological places of interest detected in Altai and Tian Shan.
The model initializes with the attractor places randomly located in both areas: Altai (three attractor places) and Tian Shan (x 3).

Environment initialization (scenarios): The model performs four distinct theoretical climate scenarios, which can be selected using the chooser in the model interface. In addition to the climate temperature, the scenarios differ in the behavioral patterns of humans, specifically in terms of energy expenditure, body temperature, and resource consumption. However, the cooperation model remains without any changes in all the scenarios.

Agents initialization (humans): Two groups of human agents are initially located in the regions of Altai and Tian Shan. Human agents start with random ages. The population size can be preconfigured using the `nHominins` slider. By using the `prob-cooperation` parameter to determine the percentage of cooperation, humans have the ability to choose an initial strategy to adopt at the beginning of the simulation, with the flexibility to change strategies during the simulation depending on different factors (see *Cooperation Model section*).

Agents initialization (attractor place): attractor places represent places where individuals can seek refuge and protection from the cold. These locations are scattered throughout Altai and Tian Shan. To aid visual differentiation, each area has attractors assigned a unique color.

For the model, each step of the simulation is equivalent to a period of 1 month. The duration of the simulation is limited to 1200 months (the equivalent of 100 years).

Submodels

Climate scenarios

We use the climate estimation proposed by Glantz et al. [9] for the glacial and interglacial periods in the Tian Shan and Altai areas (refer to Table 2 from Glantz et al., 2018). Data and temperatures were collected by selecting the Community Climate System Model (CCSM4) for the Last Glacial Maximum (26.5-19 ka), and the last interglacial (MIS 5e ca. 125,000 years ago) [10].

In our study, we incorporate seasonality into the model by updating the climate data every three months. We first adopt the climate estimation method proposed by Glantz et al. (2018) for the model setup. Then, we select the variables minimum

temperature (°C), maximum temperature (°C), and average temperature for each month for both the Altai and Tian Shan regions. To integrate seasonality into the model, we calculate the total mean for each three months to represent the results for every season. Data variables were collected and downloaded from the WordClim dataset (www.worldclim.org) [11] [12].

The model performs four distinct theoretical climate scenarios, which can be selected using the chooser in the model interface, as can be seen in **Table 4**.

**setup**			
**Scenarios**	**BioClim variables**	**Tian Shan**	**Altai**
1	Annual Mean Temperature (glacial and interglacial)	6 ºC	-4 ºC
2	Mean temperature of the coldest quarter (glacial)	-7 ºC	-21.5 ºC
3	Mean temperature of the coldest quarter (interglacial)	-9.6 ºC	-20 ºC
4	Mean temperature of warmest (Interglacial)	25 ºC	19.4 ºC
**go**			
**Scenarios**	**seasonality**	Tian Shan	Altai
1	winter	-0.98	-16.4
	spring	11.26	2.60
	summer	24.42	19.37
	autumn	12.35	3.97
2/3	winter	-7	-20
	spring	7.29	-4.42
	summer	19.20	11.54

	autumn	4.95	-2.92
4	winter	3.67	-10.05
	spring	16.64	9.83
	summer	31.59	27.18
	autumn	18.76	10.88

Table 4. Set up and go of the scenarios with the mean temperatures selected for the model based on Table 2 from Glantz et al. (2018), with modification in Scenario 1 by calculating the mean of both glacial and interglacial.

The scenarios have been designed to explore the response to humans in an environment with variable climate temperatures. A description of each scenario can be seen in **Table 5**.

Scenarios	Description
Scenario 1	Total of the means of annual temperature in Cº for glacial and interglacial scenarios
Scenario 2	Mean temperature of the coldest quarter in the Glacial Scenario
Scenario 3	Mean temperature of the coldest quarter in the Interglacial Scenario
Scenario 4	Mean temperature of the warmest quarter in the Interglacial Scenario

Table 5. Brief description of each scenario (more details in the main text).

Due to the divergence in the climate temperature in the two areas, Altai and Tian Shan are represented differently, with the Altai area having more extreme climate conditions than the Tian Shan area.

Energy gain and loss

All individuals begin with a maximum energy level of 100 %. The energy level can be adjusted using the max-energy slider, which describes the percentage of the energy of humans. The energy is spent differently, and it will vary depending on the location and the scenarios. For example, we assume that individuals are constantly spending energy at each timestep, although the amount of energy spent varies given the perception of the risk and the climate conditions. While energy consumption can be slowed down, it cannot be restored unless they are near attractor places where humans can recover energy by consuming additional resources. In addition, individuals may also consume extra energy obtained from attractor places. These places not only provide energy but also serve as shelter from extreme cold conditions for the population.

The rate of energy is also influenced by the level of perceived risk and the distance of the attractor places. In the model, in areas with more resources or attractor places, individuals assume a lower risk due to a better strategy of distribution of resources among the population. When individuals do not perceive a significant risk, the energy decreases at a much slower rate. Conversely, when individuals are located in a place with limited resources, the perceived human risk tends to increase. The energy decreases at a faster rate compared to situations with a lower perceived risk. When this perception of the risk exceeds 80%, the energy decreases even further. It is significant to notice that Individuals can perish if they deplete all their energy and the energy level drops to 0. Thus, individuals located in attractor places are expected to expend less energy compared to those in non-attractor places, as we assume that attractor locations provide shelter and a sense of safety and security. This difference in energy expenditure between the attractor and

non-attractor places is a crucial aspect of the model, as it reflects the adaptive behaviors of individuals seeking out favorable environments for resource acquisition and protection.

Tian Shan					
**Factors**		**Scenario 1**	**Scenario 2**	**Scenario 3**	**Scenario 4**
`energy`	safe place	0.01	0.02	0.02	0.01
	no safe place	0.1	0.2	0.2	0.11
`risk` (increase or decrease)	safe place	- 0.1	- 0.2	- 0.2	- 0.1
	no safe place	+ 0.1	+ 0.4	+ 0.4	+ 0.1
**Altai**					
`energy`	safe place	0.015	0.025	0.025	0.01
	no safe place	0.15	0.3	0.3	0.11
`risk` (increase or decrease)	safe place	- 0.15	- 0.3	- 0.3	- 0.1
	no safe place	+ 0.15	+ 0.5	+ 0.5	+ 0.1

Table 6. The distribution of energy spent per time step and risk varies across scenarios in different areas. The location of human agents is crucial in determining the energy expenditure and level of risk, depending on whether the area is safe or unsafe.

Body temperature

Human agents start with a body temperature of 37 ºC [13]. In extreme scenarios, we add the possibility of humans dying of hypothermia if they are not in a safe place for an extended period. When they can find a place, the body temperature increases with each step. Consequently, they must locate attractor places within a 30 km radius, providing shelter from the cold for individuals.

We also calculate the loss of body temperature:

$$\Delta T_b = (T_b - T_c) \cdot CR$$

Where ΔT_b is the result of the difference between the previous body temperature and the body temperature for each time-step; T_b is the body temperature at a given time-step; T_c is the climate temperature for each scenario in Altai or TianShan; and is CR the cooling-rate. The `cooling-rate` defines how fast a body loses temperature and is defined differently between scenarios. For more extreme scenarios, `Cooling-rate` the loss of temperature is faster than for non-extreme scenarios. In that sense, an individual can die of hypothermia when the body temperature is below 30 degrees. The individual's normal body temperature can resume once they return to a safe location.

Cooperation

The model uses an evolutionary model proposed by Henrich et al. [14], [15] to explore cooperation dilemmas in diverse climate scenarios. For the authors, the mechanism of conformist transmission allows cooperation to be maintained without the need for extensive punishment. In other words, the cost of cooperation does not

require a high punishment rate to stabilize the population when the population adopts common behavior.

The equation can be described as follows [15]:

$$\Delta p_0 = p_0(1 - p_0)[(1 - \alpha)\, \beta\, (b_c - b_d) + \alpha\, (2p_0 - 1)]$$

Where the variable Δp_0 represents the frequency of cooperators in the population; the parameter α is the strength of the conformist transmission; $(b_c - b_d)$ are the payoff of cooperators and defectors.

$b_c - b_d$ is given by the following equation:

$$b_c = (1 - e)\, (p_0\, B\, (1 - e) - C + e\, (p_0 B - N p_1 \rho)),$$

$$b_d = (1 - e)(p_0 B - N p_1 \rho)\, ,$$

$$\Delta b_0 = b_c - b_d = (1 - e)(N p_1\, (1 - e)\rho - C).$$

Where N is the number of individuals; b_c is the payoff of cooperation, while b_d is the payoff of defectors; e is the probability of failing in cooperation; C is the cost that cooperators assume to contribute; B is the benefit divided by all groups.

The model incorporates the cooperation strategy based on three key premises: a) the evolutionary framework proposed by the evolutionary model, b) the probability of individuals cooperating or defecting and c) the distribution and consumption of

resources between cooperators and defectors. A detailed list of factors for the cooperation model can be listed in **Table 7**.

**Factors**	**Overview description**
`cooperate?`	Defines if individuals are cooperators or non-cooperators/defectors. True if humans cooperate. False if humans do not cooperate. Cooperation is distinguished by colors: Humans with green color are cooperators and red color are defectors. It is defined as a boolean (true or false).
`cooperative?`	Count humans with cooperation as true.
`no cooperative?`	Count humans with cooperation as false.
`prob-cooperation`	Defines the probability of cooperation for humans at the beginning of the simulation. Default level: 0.20.
`cost-cooperation`	Defines the cost of each cooperator that reverberates similarly among the group. No cooperators do not pay the cost, but they benefit equally. Default level: 10.
`punishment-cooperation`	Defines the punishment of cooperation in defectors. Default level: 7.
`prob-nocoop`	Defines the probability of no cooperation for humans. It should be a small number. Default level: 0.001.
`alpha`	Strength of conformist transmission. Range from 0 to 1. Default level: 0.20.
`pay-off tienshan`	Pay-off of the population in Tian Shan
`pay-off altai`	Pay-off of the population in Altai
`delta-p`	Rate of change of cooperative population
`radius-for-strategy`	The radius of the strategy of cooperation that will depend on how many cooperators are around the

	agent. Default level: 3.

Table 7. Factors for the cooperation model. All the factors are defined by [15]

Cooperators are represented by the color green, while defectors and non-cooperators are represented by the color red. New individuals below the age of 12 are visually represented in black, and once they reach 12, the color changes to indicate whether they become cooperators or defectors. Thus, newborn humans can decide their strategy role until they are over the age of 12. In the model, the selection of one strategy is primarily focused on the pressure of the group, instead of being conditioned by their own experience, since they can make decisions.

At the beginning of the simulation, humans may assume one role randomly, depending on the probability of cooperation that they can set up previously. Within the model, humans interact with others in order to adopt one of two different social strategies: cooperation and non-cooperation. When humans choose to cooperate, they assume trait 1, whereas non-cooperators assume trait 0.

In the model, the selection of a cooperative strategy leads to individuals consuming fewer resources as they prioritize sustainable resource use and equitable sharing, contributing to better resource management and conservation within the population [16].

Individuals can make decisions regarding cooperation, considering both the evolutionary equation and the social pressure of the group. Social pressure works similarly to real-life scenarios: if there is a high number of individuals around who are inclined to cooperate, an individual is more likely to choose cooperation as well. This selection is influenced by the presence of cooperative and non-cooperative individuals around [17]. We assume here that the behavior of the

majority can influence others to adopt either cooperative or non-cooperative options, thereby increasing the likelihood that individuals will choose the same strategy as those around them.

However, the evolutionary model barely contemplated that individuals can decide not to cooperate at certain moments. Therefore, we have implemented this aspect into the model to address this limitation by incorporating additional factors that increase the possibility of non-cooperation decisions. Humans in the simulation have the option to not cooperate based on three key assumptions: social pressure, the probability of non-cooperation (indicating a lack of interest or willingness to cooperate), and limited resources that implement competition between individuals. These factors influence the decision-making process and allow for variations in cooperative behavior throughout the simulation.

References

[1] U. Wilensky and W. Rand, *An introduction to agent-based modeling: modeling natural, social, and engineered complex systems with NetLogo*. Mit Press, 2015.

[2] Polhill, J.G., Parker, D., Brown, D., and Grimm, V., “Using the ODD Protocol for Describing Three Agent-Based Social Simulation Models of Land-Use Change.” Accessed: Oct. 07, 2022. [Online]. Available: https://www.jasss.org/11/2/3.html

[3] V. Grimm, U. Berger, D. L. DeAngelis, J. G. Polhill, J. Giske, and S. F. Railsback, “The ODD protocol: A review and first update,” *Ecological Modelling*, vol. 221, no. 23, pp. 2760–2768, Nov. 2010, doi: 10.1016/j.ecolmodel.2010.08.019.

[4] B. Müller *et al.*, “Describing human decisions in agent-based models – ODD + D, an extension of the ODD protocol,” *Environmental Modelling & Software*, vol. 48, pp. 37–48, Oct. 2013, doi: 10.1016/j.envsoft.2013.06.003.

[5] V. Grimm *et al.*, “The ODD Protocol for Describing Agent-Based and Other Simulation Models: A Second Update to Improve Clarity, Replication, and Structural Realism,” *JASSS*, vol. 23, no. 2, p. 7, 2020.

[6] D. M. Carballo, *Cooperation and collective action: archaeological perspectives*. University Press of Colorado, 2012.

[7] J. I. Santos *et al.*, “Effect of Resource Spatial Correlation and Hunter-Fisher-Gatherer Mobility on Social Cooperation in Tierra del Fuego,” *PLOS ONE*, vol. 10, no. 4, p. e0121888, Apr. 2015, doi: 10.1371/journal.pone.0121888.

[8] Wren and Costopoulos, “Does Environmental Knowledge Inhibit Hominin Dispersal?,” *Human Biology*, vol. 87, no. 3, p. 205, 2015, doi: 10.13110/humanbiology.87.3.0205.

[9] M. M. Glantz, A. Van Arsdale, S. Temirbekov, and T. Beeton, “How to survive the glacial apocalypse: Hominin mobility strategies in late Pleistocene Central Asia,” *Quaternary International*, vol. 466, pp. 82–92, Feb. 2018, doi: 10.1016/j.quaint.2016.06.037.

[10] B. L. Otto-Bliesner, S. J. Marshall, J. T. Overpeck, G. H. Miller, A. Hu, and CAPE LAST INTERGLACIAL PROJECT MEMBERS, “Simulating Arctic Climate Warmth and Icefield

Retreat in the Last Interglaciation," *Science*, vol. 311, no. 5768, pp. 1751–1753, Mar. 2006, doi: 10.1126/science.1120808.

[11] R. J. Hijmans, S. E. Cameron, J. L. Parra, P. G. Jones, and A. Jarvis, "Very high resolution interpolated climate surfaces for global land areas," *International Journal of Climatology*, vol. 25, no. 15, pp. 1965–1978, 2005, doi: 10.1002/joc.1276.

[12] S. E. Fick and R. J. Hijmans, "WorldClim 2: new 1-km spatial resolution climate surfaces for global land areas," *International Journal of Climatology*, vol. 37, no. 12, pp. 4302–4315, 2017, doi: 10.1002/joc.5086.

[13] P. Petrone, J. A. Asensio, and C. P. Marini, "Management of accidental hypothermia and cold injury," *Current Problems in Surgery*, vol. 51, no. 10, pp. 417–431, Oct. 2014, doi: 10.1067/j.cpsurg.2014.07.004.

[14] J. Henrich, "Cultural transmission and the diffusion of innovations: Adoption dynamics indicate that biased cultural transmission is the predominate force in behavioral change," *American Anthropologist*, vol. 103, no. 4, pp. 992–1013, 2001.

[15] J. Henrich and R. Boyd, "Why People Punish Defectors: Weak Conformist Transmission can Stabilize Costly Enforcement of Norms in Cooperative Dilemmas," *Journal of Theoretical Biology*, vol. 208, no. 1, pp. 79–89, Enero 2001, doi: 10.1006/jtbi.2000.2202.

[16] A. Angourakis, J. I. Santos, J. M. Galán, and A. L. Balbo, "Food for all: An agent-based model to explore the emergence and implications of cooperation for food storage," *Environmental Archaeology*, vol. 20, no. 4, pp. 349–363, Nov. 2015, doi: 10.1179/1749631414Y.0000000041.

[17] C. Hilbe, K. Chatterjee, and M. A. Nowak, "Partners and rivals in direct reciprocity," *Nat Hum Behav*, vol. 2, no. 7, Art. no. 7, Jul. 2018, doi: 10.1038/s41562-018-0320-9.
