# Supplementary material for: The Impact of Cooperation Under Climate Constraints: An Agent-Based Model for Exploring Paleolithic Behavioral Adaptations in the Inner Asian Mountain Corridor
Source: J Archaeol Method Theory. 2025 Sep 20;33(1):8. doi: 10.1007/s10816-025-09739-z (PMC12450231; doi:10.1007/s10816-025-09739-z)
Supplement: Supplementary file 5 — (PDF 2.12 KB) [file 10816_2025_9739_MOESM5_ESM.pdf]

Supplementary Material

List of extra figures in the results section

Probability of cooperation

Scenario 1: Average annual glacial-interglacial temperatures

In the case of Tian Shan**,** we detect a significant increase in the number of cooperators when the probability of cooperation is equal to or greater than 50% **(Fig. S1-S2)**. Conversely, when the probability is reduced to 20%, cooperators fail to increase in number within the population during the simulation, except when alpha is lower **(Fig. S3)**. Despite the increase of non-cooperators and defectors when the probability is set to 20 %, individuals display lower chances of long-term survival and population increase compared to scenarios with a higher probability of cooperators.

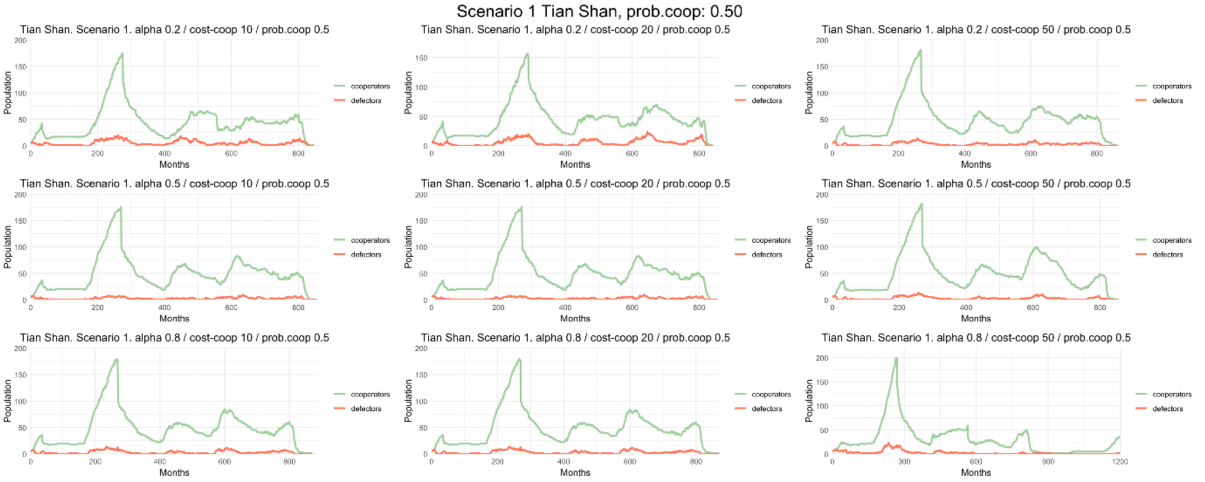

Figure S1. Observed Patterns in Scenario 1 (Tian Shan) with 50% Cooperation Probability. The graphs display population dynamics over time (measured in months) for Scenario 1, where the probability of cooperation is 50%. Each graph corresponds

to a different simulation, with varying levels of cooperation cost and the alpha parameter. These variations allow for a detailed analysis of how changes in these factors influence population trends and cooperative behaviors in the simulated environment.

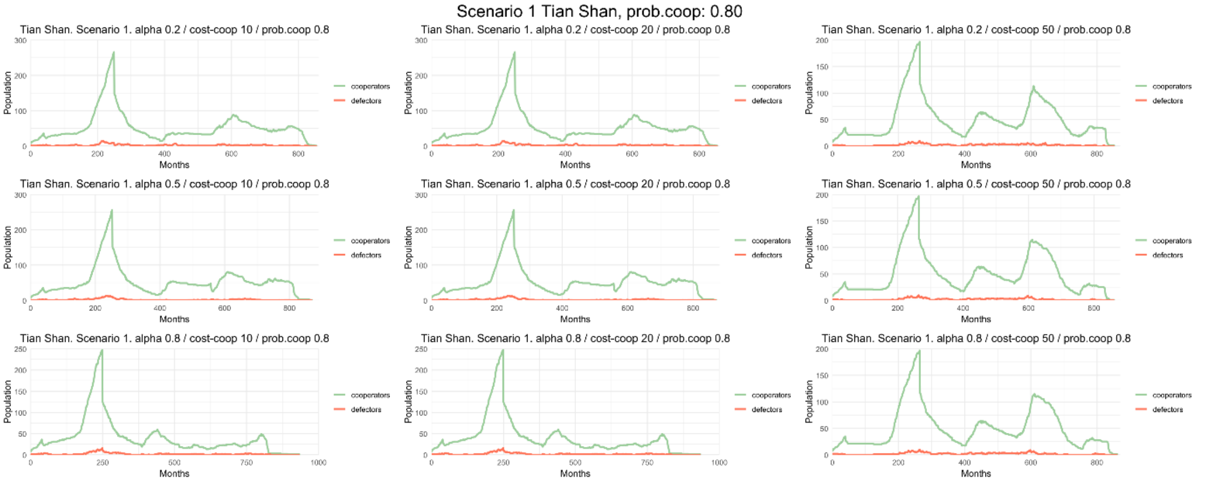

Figure S2. Observed Patterns in Scenario 1 (Tian Shan) with 80% Cooperation Probability. The graphs display population dynamics over time (measured in months) for Scenario 1, where the probability of cooperation is set to 80%. Each graph corresponds to a different simulation, with varying levels of cooperation cost and the alpha parameter. These variations allow for a detailed analysis of how changes in these factors influence population trends and cooperative behaviors in the simulated environment.

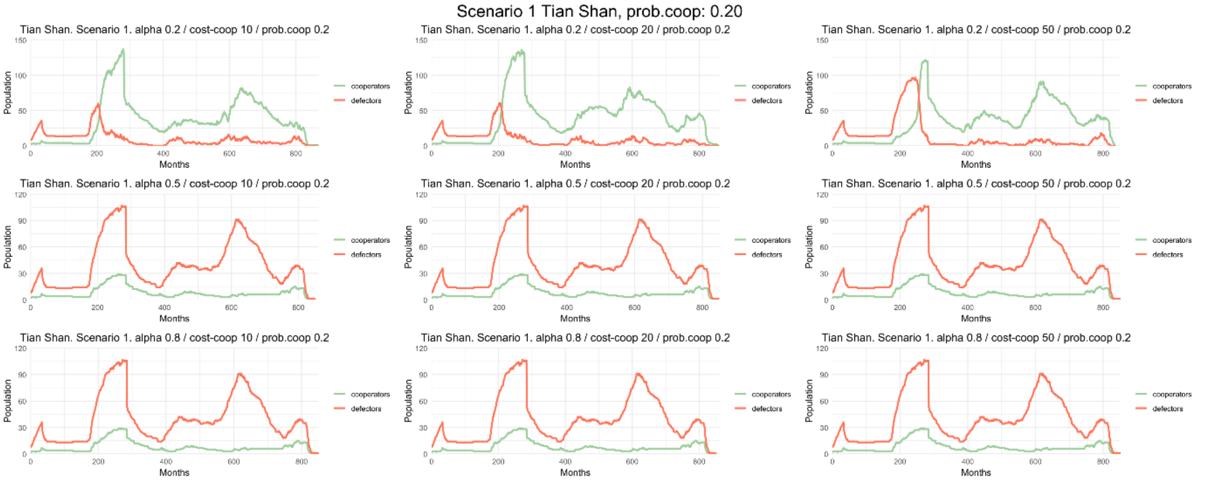

Figure S3. Observed Patterns in Scenario 1 (Tian Shan) with 20% Cooperation Probability. The graphs display population dynamics over time (measured in months) for Scenario 1, where the probability of cooperation is set to 20%. Each graph corresponds to a different simulation, with varying levels of cooperation cost and the alpha parameter. These variations allow for a detailed analysis of how changes in these factors influence population trends and cooperative behaviors in the simulated environment.

In the Altai region**,** Scenario 1 displays a similar pattern comparable with fluctuations in population size attributed to varying climate temperatures. We observed minimal changes in population size and the presence of cooperators when the probability of cooperation is set to 20 %, 50 % and 80 %, with a slightly higher variation of the persistence of cooperators observed when the probability is 80 % **(Figs. S4, S5, S6).** As seen in Tian Shan, the scenario with a 20 % probability of cooperation reveals a persistent presence of cooperators, increasing in numbers during the simulation.

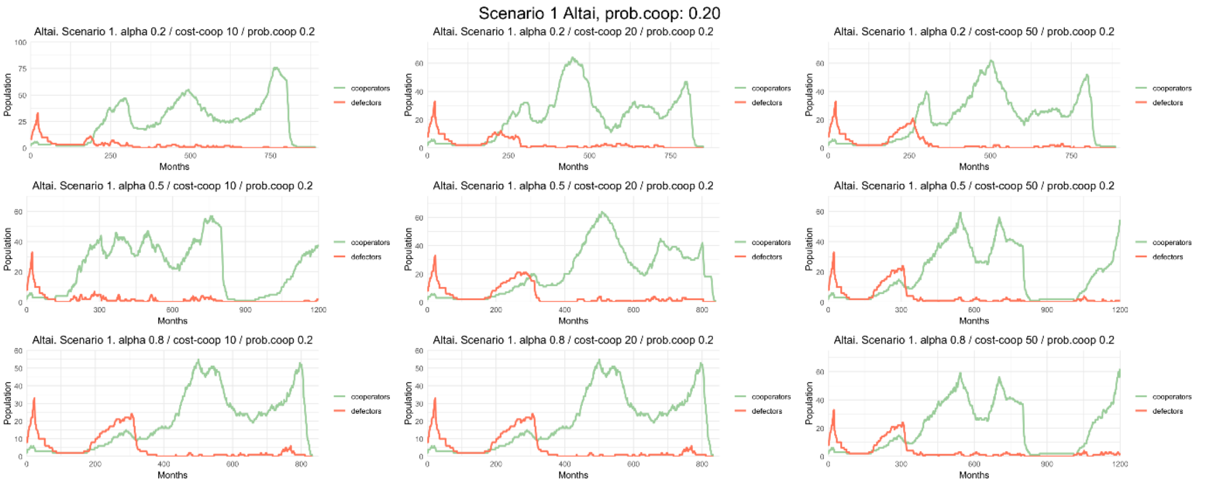

Figure S4. Observed Patterns in Scenario 1 (Altai) with 20% Cooperation Probability. The graphs display population dynamics over time (measured in months) for Scenario 1, where the probability of cooperation is set to 20%. Each graph corresponds to a different simulation, with varying levels of cooperation cost and the alpha parameter.

These variations allow for a detailed analysis of how changes in these factors influence population trends and cooperative behaviors in the simulated environment.

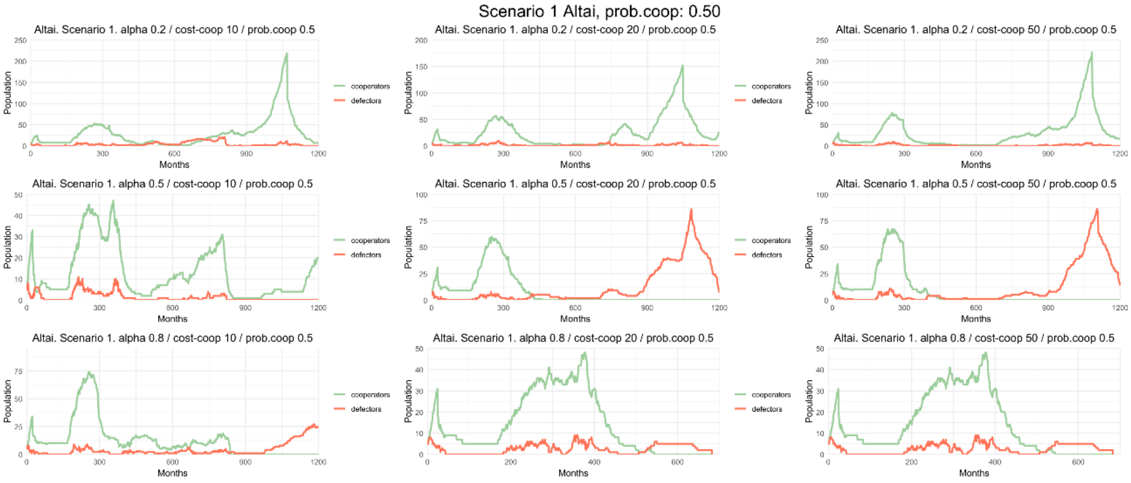

Figure S5. Observed Patterns in Scenario 1 (Altai) with 50% Cooperation Probability. The graphs display population dynamics over time (measured in months) for Scenario 1, where the probability of cooperation is set to 50%. Each graph corresponds to a different simulation, with varying levels of cooperation cost and the alpha parameter. These variations allow for a detailed analysis of how changes in these factors influence population trends and cooperative behaviors in the simulated environment.

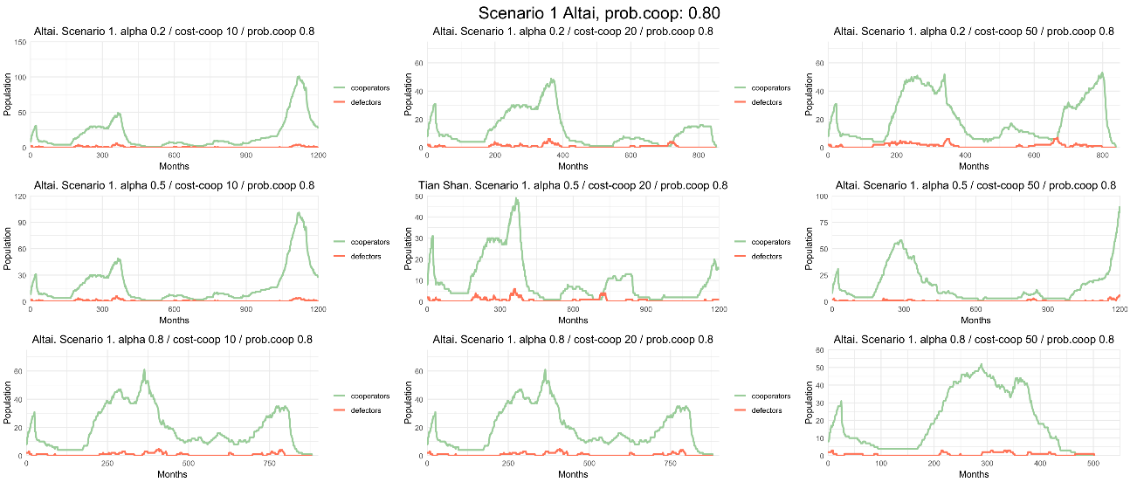

Figure S6. Observed Patterns in Scenario 1 (Altai) with 80% Cooperation Probability. The graphs display population dynamics over time (measured in months) for Scenario 1, where the probability of cooperation is set to 80%. Each graph corresponds to a different simulation, with varying levels of cooperation cost and the alpha parameter. These variations allow for a detailed analysis of how changes in these factors influence population trends and cooperative behaviors in the simulated environment.

Scenarios 2 (glacial high seasonality) and 3 (interglacial high seasonality)

We conducted separate simulations for Scenarios 2 and 3; however, we did not observe any discernible changes attributed to the minor differences in climate temperatures between glacial and interglacial periods in either the Altai or Tian Shan regions.

In both Tian Shan Scenarios 2 and 3, we detect a slightly similar pattern emerging when the climate temperature becomes more extreme, especially when the probability of cooperation is below 50 % **(Fig. S7)**. An increase in non-cooperators and defectors is observed but due to the extreme temperature in the case of the Altai, the total population does not increase **(Fig. S8)**.

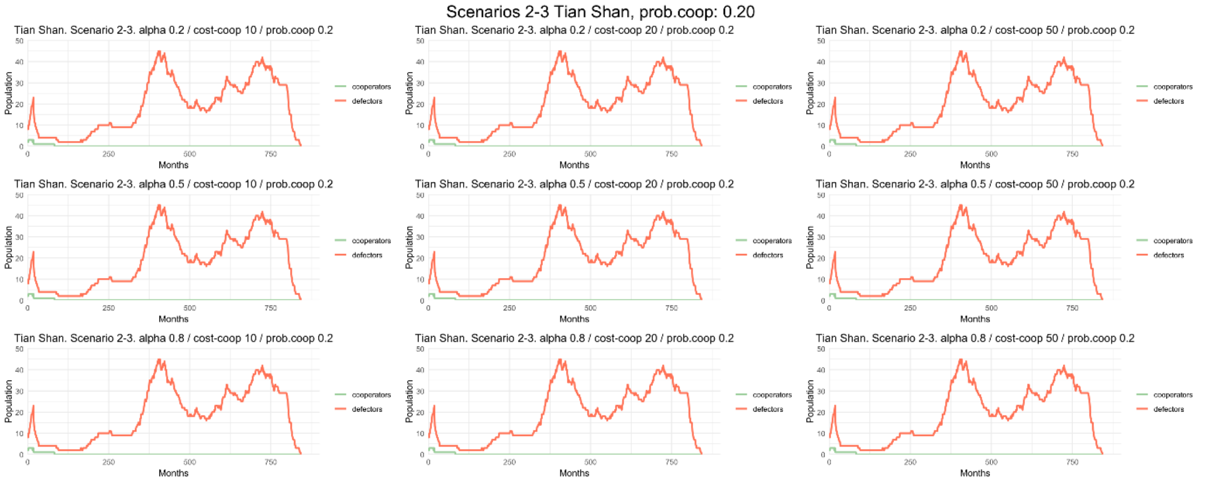

Figure S7. Patterns observed in Scenarios 2-3 (Tian Shan) with 20% Cooperation Probability. The graphs display population dynamics over time (measured in months)

for Scenarios 2-3, where the probability of cooperation is set to 20%. Each graph corresponds to a different simulation, with varying levels of cooperation cost and the alpha parameter. These variations allow for a detailed analysis of how changes in these factors influence population trends and cooperative behaviors in the simulated environment.

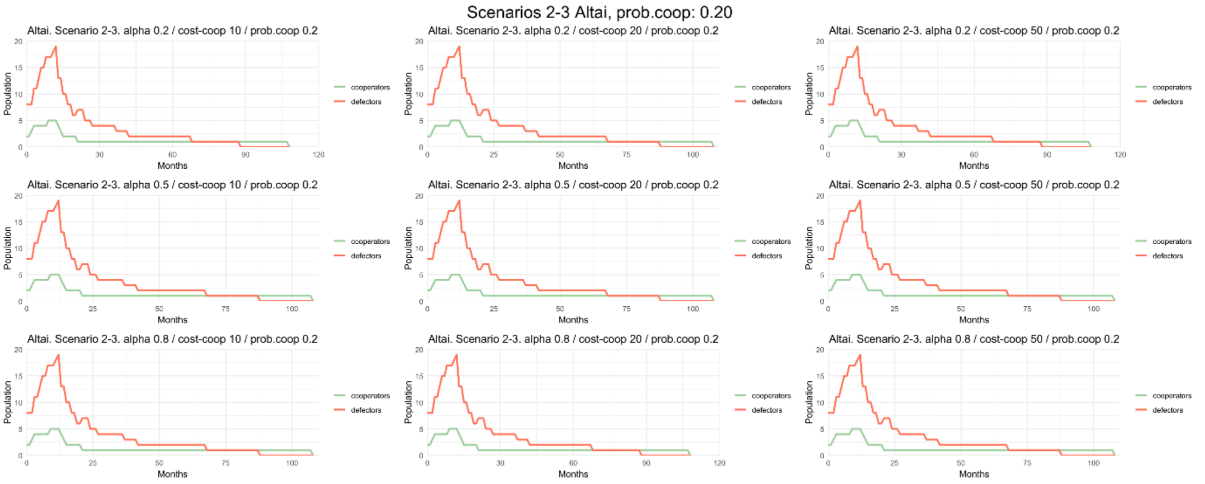

Fig. S8. Patterns observed in Scenario 2-3 (Altai) with 20% Cooperation Probability. The graphs display population dynamics over time (measured in months) for Scenarios 2-3, where the probability of cooperation is set to 20%. Each graph corresponds to a different simulation, with varying levels of cooperation cost and the alpha parameter. These variations allow for a detailed analysis of how changes in these factors influence population trends and cooperative behaviors in the simulated environment.

In contrast, when the probability of cooperation exceeds 50 %, the Altai and Tian Shan show different patterns in terms of survival **(Figs. S9-S10)**. We observe a higher competition between cooperators and non-cooperators in the Altai than in the Tian Shan, especially when the cost of cooperation increases. Under these conditions, the probability of survival decreases in cooperators. However, cooperators tended to exhibit higher survival rates compared to non-cooperators throughout the simulation.

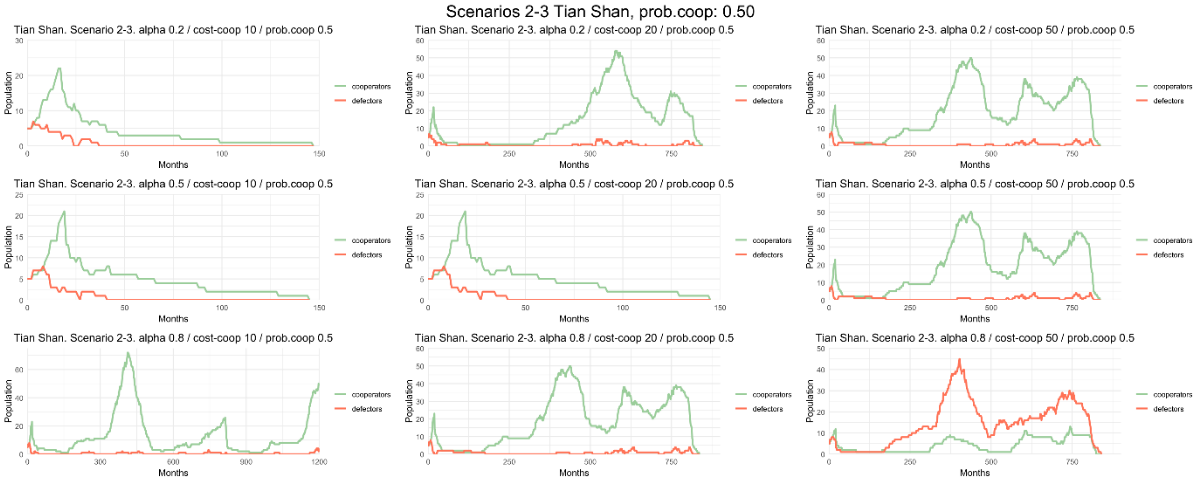

Figure S9. Patterns observed in Scenarios 2-3 (Tian Shan) with 50% Cooperation Probability. The graphs display population dynamics over time (measured in months) for Scenarios 2-3, where the probability of cooperation is set to 50%. Each graph corresponds to a different simulation, with varying levels of cooperation cost and the alpha parameter. These variations allow for a detailed analysis of how changes in these factors influence population trends and cooperative behaviors in the simulated environment.

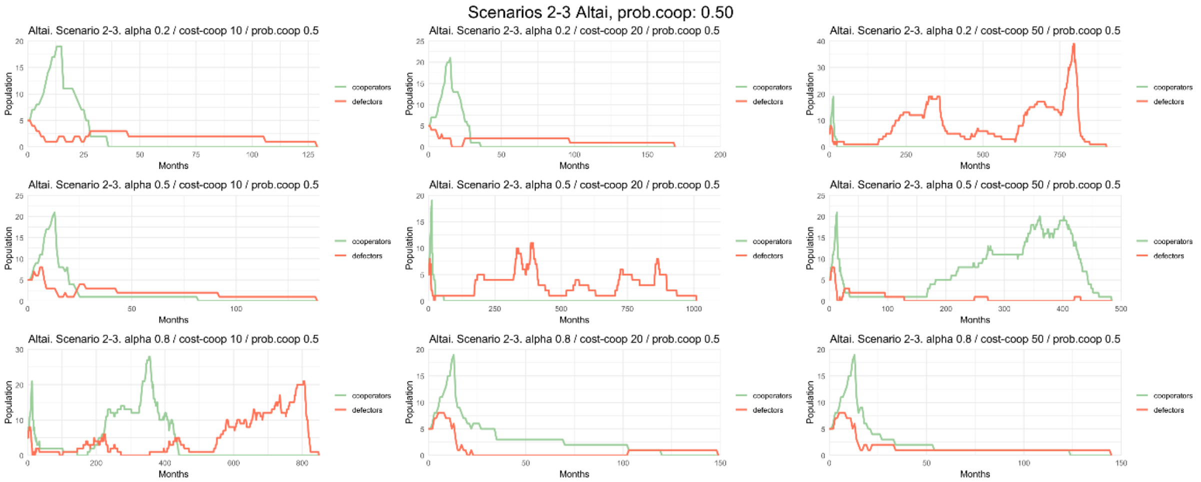

Figure S10. Patterns observed in Scenarios 2-3 (Altai) with 50% Cooperation Probability. The graphs display population dynamics over time (measured in months) for Scenarios 2-3, where the probability of cooperation is set to 50%. Each graph corresponds to a different simulation, with varying levels of cooperation cost and the alpha parameter. These variations allow for a detailed analysis of how changes in these factors influence population trends and cooperative behaviors in the simulated environment.

It is worth mentioning here that the climate temperature and environmental conditions in the Altai presented a higher level of hostility compared to the Tian Shan. Under these conditions, we identified a higher prevalence of survival when the probability of cooperation was set to 80% in Tian Shan **(Fig. S11)**. Surprisingly, the higher prevalence and the increase of population in Altai occurred when the cost of cooperation and alpha was lower **(Fig. S12).**

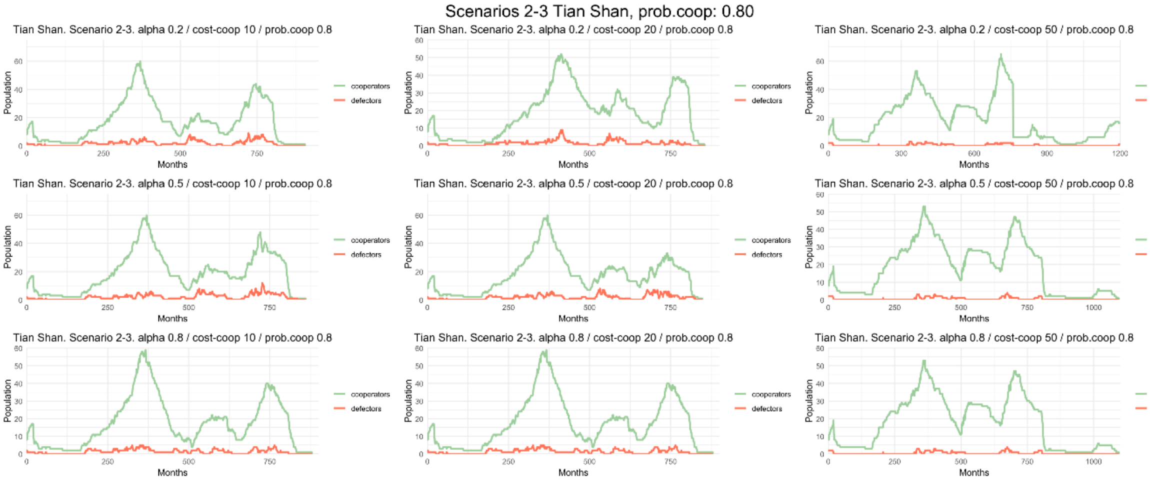

Figure S11. Patterns observed in Scenarios 2-3 (Tian Shan) with 80% Cooperation Probability. The graphs display population dynamics over time (measured in months) for Scenarios 2-3, where the probability of cooperation is set to 80%. Each graph corresponds to a different simulation, with varying levels of cooperation cost and the alpha parameter. These variations allow for a detailed analysis of how changes in

these factors influence population trends and cooperative behaviors in the simulated environment.

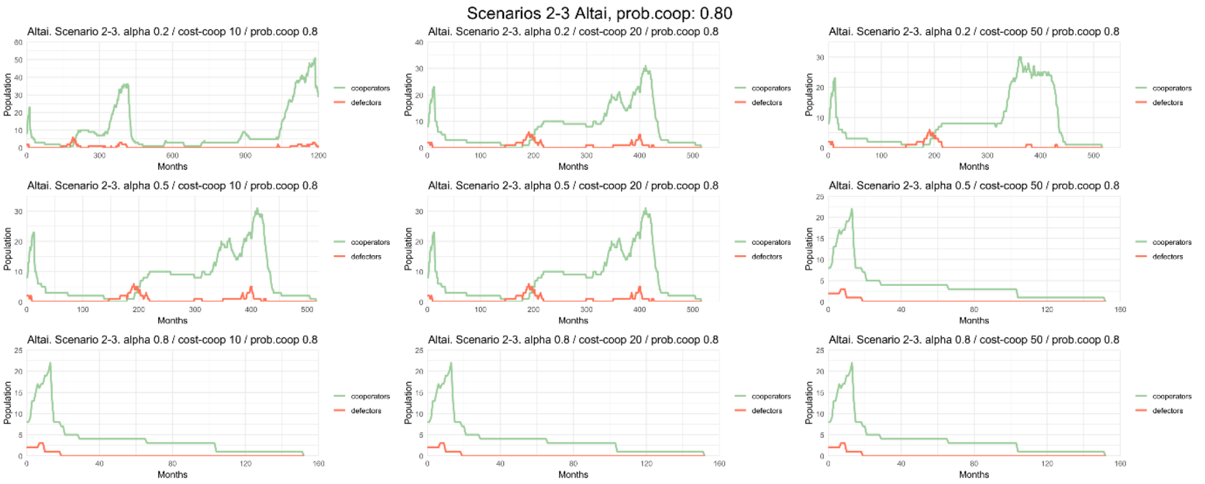

Figure S12. Patterns observed in Scenarios 2-3 (Altai) with 80% Cooperation Probability. The graphs display population dynamics over time (measured in months) for Scenarios 2-3, where the probability of cooperation is set to 80%. Each graph corresponds to a different simulation, with varying levels of cooperation cost and the alpha parameter. These variations allow for a detailed analysis of how changes in these factors influence population trends and cooperative behaviors in the simulated environment.

The contrasting results between the Tian Shan and Altai scenarios underscore the sensitivity of cooperative behaviors to varying environmental conditions and climate temperatures. In any case, in both the Altai and Tian Shan, the population grows less than in scenarios 1 and 4.

Scenario 4: warmest interglacial

This scenario corresponds to a simulation involving the warmest temperatures in both the Altai and Tian Shan regions, combining aspects of scenarios 1 and 2, and specifically focusing on resource consumption and energy usage. The aim was to test a potential warm scenario with limited resources but a non-significant loss of energy compared to scenarios 2 and 3.

In both regions (Tian Shan and Altai), we observed similar patterns in Scenario 4, mostly when the probability of cooperation is set to 80%. This could be attributed to a scenario where there are no significant differences in extreme temperature between them.

In the Tian Shan, Scenario 4 shows an increase in population size when the probability of cooperation was set to 50% and 80% **(Figs. S13-S14)**, whereas the number of both cooperators and defectors is relatively low when the probability is set to 20% **(Fig. S15)**. Despite being a scenario without extreme temperatures, we can see cooperators struggling to maintain large populations when the probabilities are lower (20%) unlike scenario 1 in the Tian Shan where cooperators outnumber non-cooperators by a majority.

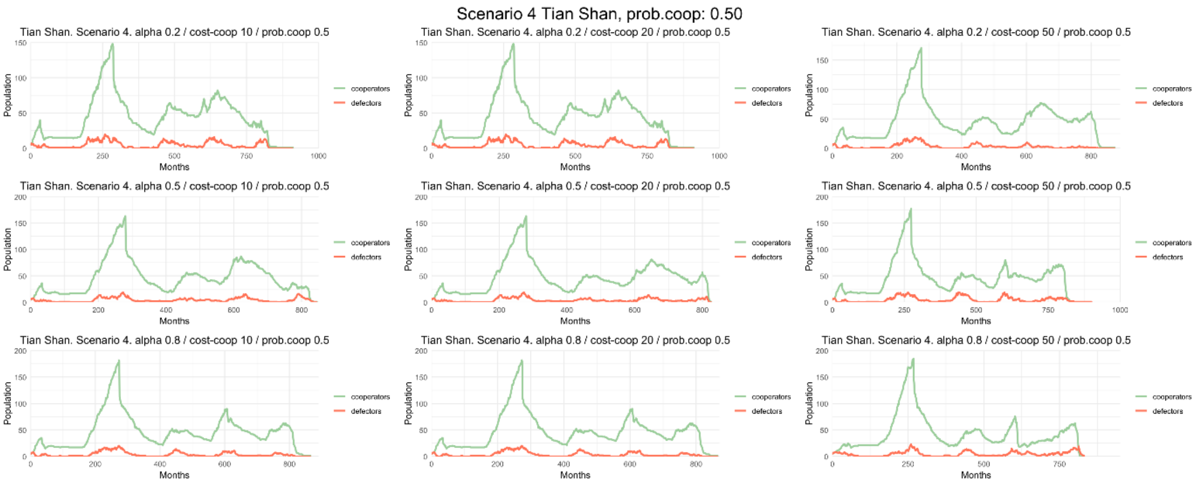

Figure S13. Patterns observed in Scenario 4 (Tian Shan) with 50% Cooperation Probability. The graphs display population dynamics over time (measured in months) for Scenario 4, where the probability of cooperation is set to 50%. Each graph corresponds to a different simulation, with varying levels of cooperation cost and the alpha parameter. These variations allow for a detailed analysis of how changes in these factors influence population trends and cooperative behaviors in the simulated environment.

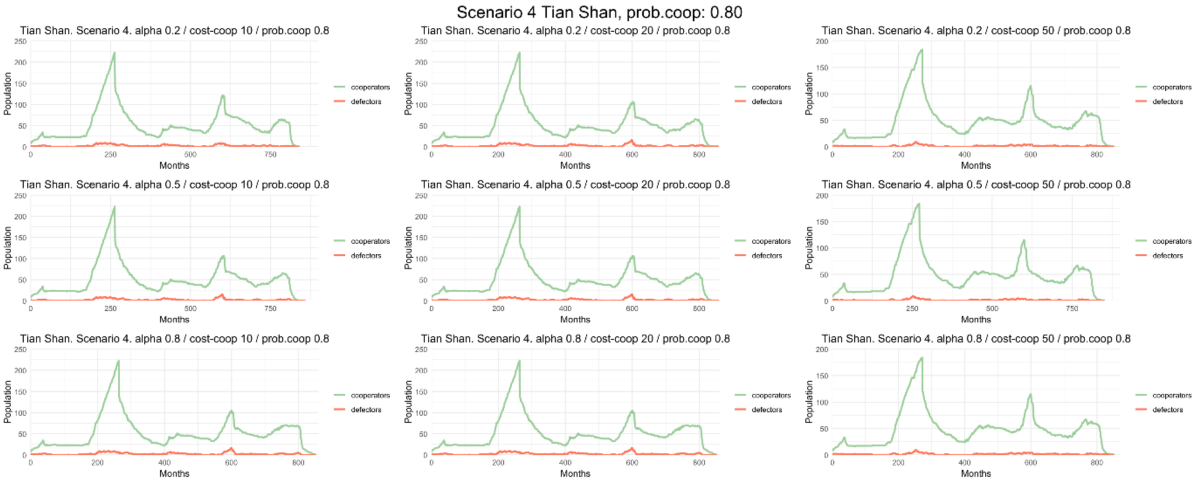

Figure S14. Patterns observed in Scenario 4 (Tian Shan) with 80% Cooperation Probability. The graphs display population dynamics over time (measured in months) for Scenario 4, where the probability of cooperation is set to 80%. Each graph corresponds to a different simulation, with varying levels of cooperation cost and the alpha parameter. These variations allow for a detailed analysis of how changes in these factors influence population trends and cooperative behaviors in the simulated environment.

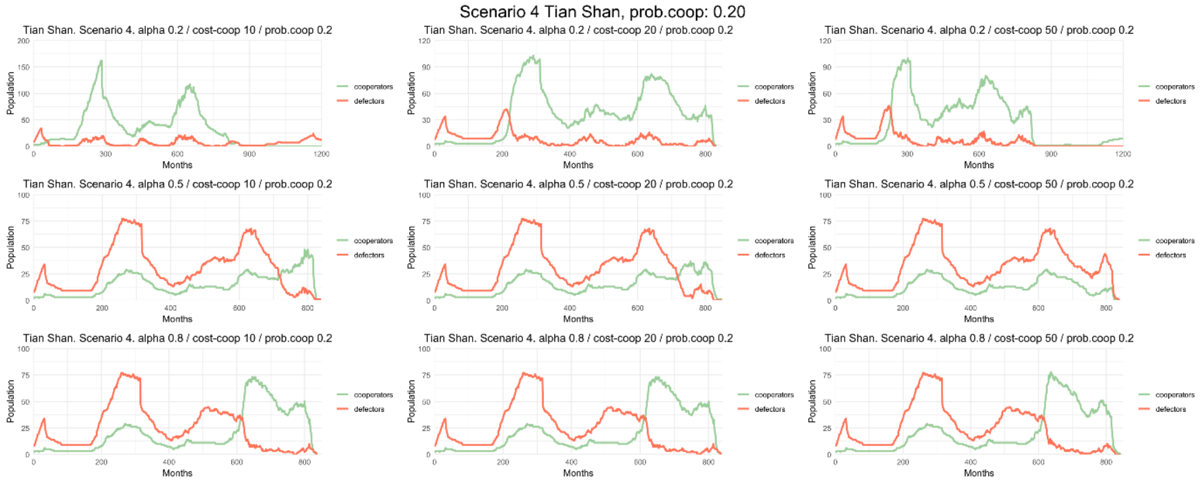

Figure S15. Patterns observed in Scenario 4 (Tian Shan) with 20% Cooperation Probability. The graphs display population dynamics over time (measured in months) for Scenario 4, where the probability of cooperation is set to 20%. Each graph corresponds to a different simulation, with varying levels of cooperation cost and the alpha parameter. These variations allow for a detailed analysis of how changes in these factors influence population trends and cooperative behaviors in the simulated environment.

When the probability of cooperation was set to 80%, the Altai followed a similar pattern to the Tian Shan, albeit with a higher increase in population size **(Fig. S16)**. When the probability of cooperation was 20%, the survival rate of cooperators in the Altai was higher than in the Tian Shan **(Fig. S17)**, but they hardly differed when the probability of cooperation was set to 50% in both regions, except when the alpha is higher in the Altai Scenario **(Fig. S18)**.

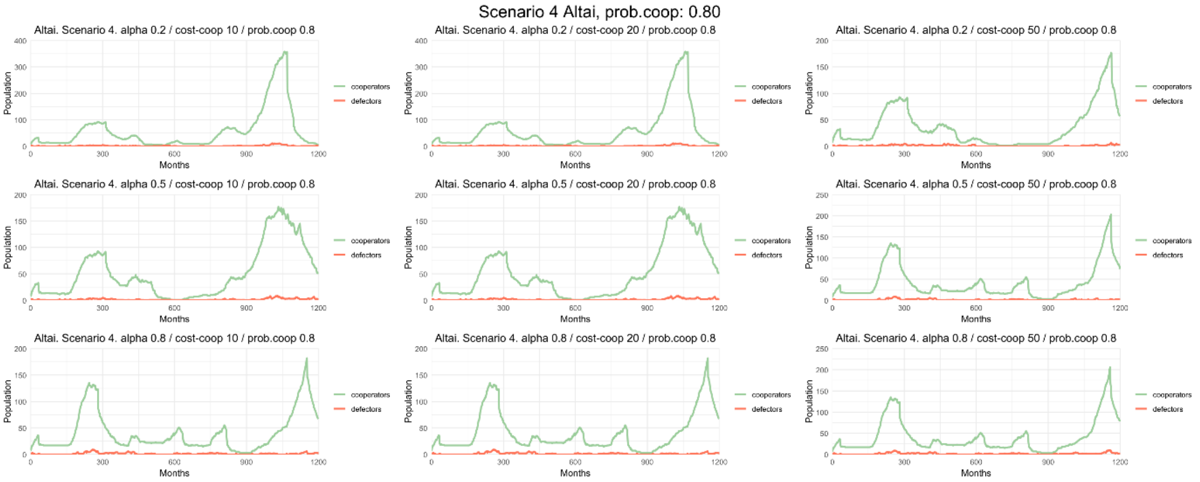

Figure S16. Patterns observed in Scenario 4 (Altai) with 80% Cooperation Probability. The graphs display population dynamics over time (measured in months) for Scenario 4, where the probability of cooperation is set to 80%. Each graph corresponds to a different simulation, with varying levels of cooperation cost and the alpha parameter. These variations allow for a detailed analysis of how changes in these factors influence population trends and cooperative behaviors in the simulated environment.

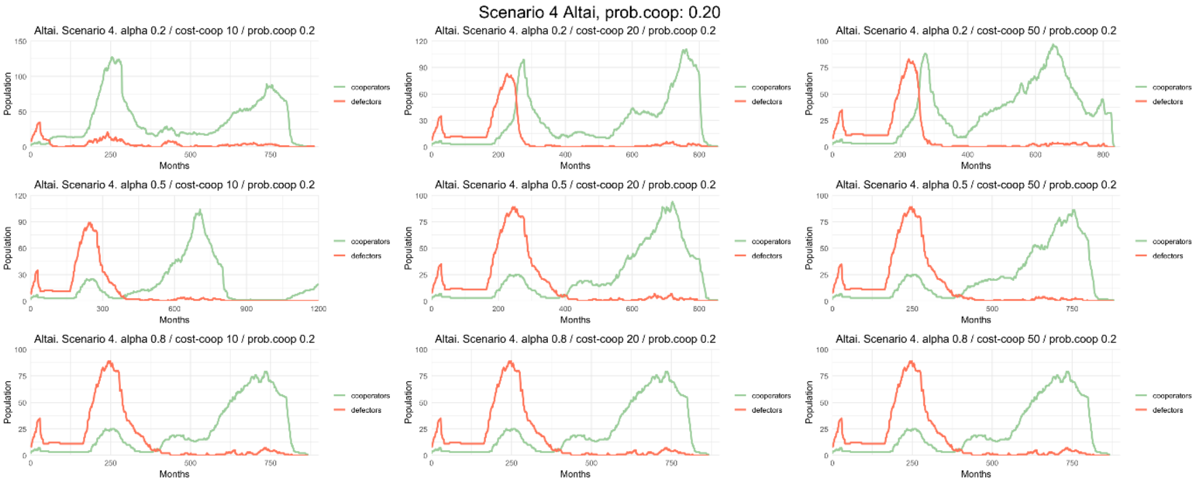

Figure S17. Patterns observed in Scenario 4 (Altai) with 20% Cooperation Probability. The graphs display population dynamics over time (measured in months) for Scenario 4, where the probability of cooperation is set to 20%. Each graph corresponds to a different simulation, with varying levels of cooperation cost and the alpha parameter.

These variations allow for a detailed analysis of how changes in these factors influence population trends and cooperative behaviors in the simulated environment.

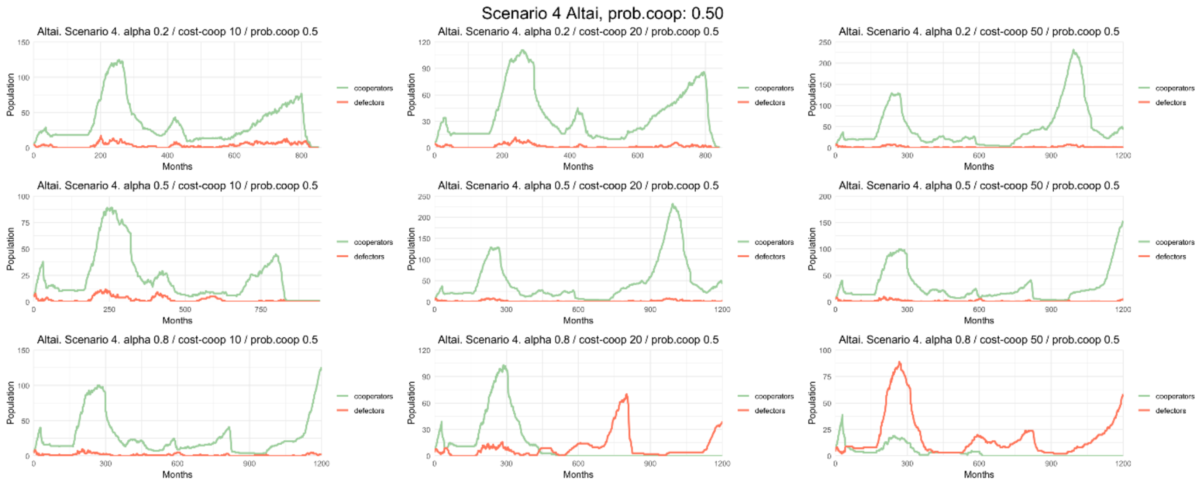

Figure S18. Patterns observed in Scenario 4 (Altai) with 50% Cooperation Probability. The graphs display population dynamics over time (measured in months) for Scenario 4, where the probability of cooperation is set to 50%. Each graph corresponds to a different simulation, with varying levels of cooperation cost and the alpha parameter. These variations allow for a detailed analysis of how changes in these factors influence population trends and cooperative behaviors in the simulated environment.
